# Dynamic monitoring of PD‐L1 and Ki67 in circulating tumor cells of metastatic non‐small cell lung cancer patients treated with pembrolizumab

**DOI:** 10.1002/1878-0261.13317

**Published:** 2022-12-16

**Authors:** Maria Spiliotaki, Christiana Michael Neophytou, Paris Vogazianos, Ioannis Stylianou, Gregoria Gregoriou, Andreas Ioannou Constantinou, Constantinos Deltas, Haris Charalambous

**Affiliations:** ^1^ Laboratory of Cancer Biology and Chemoprevention, Department of Biological Sciences University of Cyprus Nicosia Cyprus; ^2^ biobank.cy Center of Excellence in Biobanking and Biomedical Research, Molecular Medicine Research Center University of Cyprus Nicosia Cyprus; ^3^ European University Research Center Nicosia Cyprus; ^4^ European University of Cyprus Nicosia Cyprus; ^5^ Bank of Cyprus Oncology Centre Nicosia Cyprus; ^6^ University of Cyprus Medical School Nicosia Cyprus

**Keywords:** CTC, Ki67, NSCLC, PD‐L1, pembrolizumab, primary resistance

## Abstract

Programmed cell death protein ligand‐1 (*PD‐L1*) expression in non‐small cell lung cancer (NSCLC) tumors guides treatment selection. *PD‐L1* expression in circulating tumor cells (CTCs) may provide further information. We have explored *PD‐L1* and marker of proliferation Ki‐67 (*Ki67*; also known as *MKI67*) in CTCs in longitudinal samples of 47 advanced NSCLC patients receiving pembrolizumab. A triple immunofluorescence, against cytokeratin, PD‐L1, and Ki67, was performed on peripheral blood mononuclear cells, at baseline, post‐first cycle, post‐third, and primary resistance (PMR). Patients displaying PMR (progression at first evaluation) were classified as progressive disease (PD) and those with clinical benefit as disease control (DC). CTCs were categorized as PD‐L1high/low/medium/negative and Ki67^+^ or Ki67^−^. CTC evaluation revealed a significant increase in the PD‐L1low CTC rate at PMR compared to baseline (2.5% at baseline vs. 36.5% at PMR), whereas a reduction in the PD‐L1high CTC rate was observed (31.5% vs. 0%, respectively). Investigation of CTC status between PD and DC patients showed that PD patients more frequently increased total and PD‐L1low CTCs after first cycle compared to DC (83% of PD vs. 37% of DC and 67% of PD vs. 8% of DC, respectively). Progression‐free survival (PFS) was longer in patients with decreased total and PD‐L1low CTCs after first cycle compared to those with increased CTCs (median PFS: not reached vs. 2 months). PD‐L1^+^ patients presenting a high Ki67 index (% Ki67^+^ CTCs > 30%) before treatment had a shorter PFS compared to those with a low Ki67 (≤ 30%), and overall survival (OS) was shorter in PD‐L1^+^ patients harboring Ki67^+^ CTCs compared to those not presenting (median OS: 11.8 months vs. 33.1 months, respectively). In sequential samples of patients with a durable benefit, a low Ki67 index was observed. Our results suggest that monitoring *PD‐L1* and *Ki67* expression in CTCs of NSCLC patients treated with pembrolizumab may be predictive for pembrolizumab efficacy.

## Graphical abstract

The authors investigated the differential expression of PD‐L1 (high, medium, low, negative) and Ki67 in circulating tumor cells (CTCs) of non‐small cell lung cancer patients treated with pembrolizumab. A high percentage of Ki67^+^ CTCs (> 30%) before treatment, or an increase in total number of CTCs or in PD‐L1low CTCs after the first cycle, seems to be related to resistance to pembrolizumab.

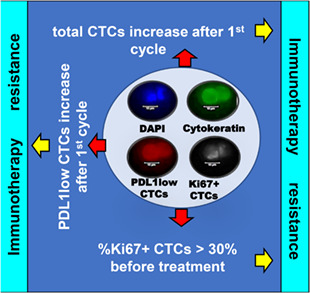

**Abbreviations**
CK: cytokeratin
CTCs: circulating tumor cells
DC: disease control
FBS: fetal bovine serum
HR: hazard ratio
ICIs: immune checkpoint inhibitors
MFI: mean fluorescence intensity
NSCLC: non‐small cell lung cancer
OS: overall survival
PBMCs: peripheral blood mononuclear cells
PBS: phosphate‐buffered saline
PD: progression disease
PD‐1: programmed cell death protein 1
PD‐L1: programmed cell death protein ligand‐1
PFS: progression‐free survival
PMR: primary resistance
RT: room temperature
TBST: tris‐buffered saline/Tween 20

## 1 Introduction

Lung cancer is the leading cause of cancer‐related death affecting over 1.8 million people worldwide every year. There is an estimated 18% survival rate beyond 5 years for all stages combined, with poor outcomes largely related to late diagnosis [1]. Recently, the use of immunotherapy primarily with immune checkpoint inhibitors (ICIs) has been increasingly applied in the treatment of lung cancer.

Immune checkpoint inhibitors have been designed to target inhibitory checkpoint molecules such as the programmed cell death protein 1 (PD‐1) and its ligand, PD‐L1. The immune response to cancer involves a complex network of cellular interactions between cytotoxic T cells, helper T cells, natural killer, and tumor cells. However, metastatic tumors have acquired mechanisms to evade immune detection and one such example is through the overexpression of PD‐L1 [2].

The promising therapeutic activity of ICIs in NSCLC has led the US Food and Drug Administration (FDA) to approve pembrolizumab, an antibody directed against the PD‐1 immune checkpoint molecule for the treatment of NSCLC patients whose tumors express PD‐L1 [3]. It has been shown that ICIs are most effective in patients with ‘inflamed tumors’ characterized by PD‐L1 expression, high proportion of tumor‐infiltrating immune cells (TILs), or presence of a strong IFN‐γ cytolytic T‐ cell signature and act by reinvigorating a pre‐existing antitumor T‐cell response [4, 5, 6].

Unfortunately, responses to ICI treatment are not ubiquitous. Interestingly, many patients experience a primary or innate resistance at first evaluation to ICI treatment [7, 8] while others obtain an initial clinical benefit before experiencing disease progression, displaying secondary, or acquired resistance [7]. Finally, only a subset of patients will exhibit durable and lasting responses, underlying the need for the discovery of reliable predictive biomarkers. Finding biomarker prognosticators of response to PD‐L1 blockade has been proven extremely challenging. Previous studies suggested that a high PD‐L1 tumor proportion score (TPS) on the initial tumor biopsy is a predictor of a positive response [3]. Despite expectations, not all patients with high PD‐L1 expression in their tumors obtain clinical benefits from ICIs, indicating that factors unrelated to PD‐L1 and PD‐1 come into play [9]. The proliferation marker Ki67 has been considered to play a prognostic role and was investigated in several cancer types including lung cancer [10, 11, 12]. Recently, tumor specimens from NSCLC patients treated with ICIs, were evaluated for PD‐L1 expression by immunohistochemistry and for a proliferative profile by RNA‐seq showing that cell proliferation is potentially a new biomarker of response to ICIs in NSCLC [13].

Liquid biopsies, such as CTCs shed from various locations of the primary and/or metastatic tumors allow a rapid and accurate identification of the *de novo* and resistant genetic alterations, as well as real‐time monitoring of treatment responses [14, 15]. The feasibility of non‐invasive analysis of PD‐L1 expression on CTCs with different techniques in advanced NSCLC has been reported [16, 17, 18, 19]. However, analysis of the results and establishment of reliable predictors of patient outcomes has been hampered by obstacles including different PD‐L1 antibodies, CTC‐enrichment methods, small number of patients in each trial, the lack of serial samples, and a short follow‐up time.

In the present study, we have explored CTC status in longitudinal samples of 47 metastatic NSCLC patients treated with pembrolizumab. We initially established a semiquantitative method to evaluate PD‐L1 expression levels and then assessed PD‐L1 and Ki67 on CTCs from the enrolled patients before treatment (baseline), at post‐first, post‐third cycle, and primary resistance. Subsequently, we compared total CTC counts, the number and percentage of PD‐L1 and Ki67 subpopulations between baseline and each timepoint. Moreover, we examined CTC changes before and after the first cycle, between patients presenting primary resistance and disease control. Finally, we investigated CTC status in serial samples of patients who experienced a durable benefit from treatment.

## 2 Materials and methods

### 2.1 Patients

Patients with metastatic NSCLC who received first‐line chemotherapy with platinum doublet, entered the ICI pembrolizumab maintenance trial (NCT02705820) if they had not progressed after 4–6 cycles of chemotherapy. The NCT02705820 trial compared maintenance pembrolizumab to best supportive care. All patients had signed written informed consent. The study methodologies conformed to the standards set by the Declaration of Helsinki and they were also approved by the Cyprus National Bioethics Committee (2019/76).

During their treatment with pembrolizumab the patients were enrolled in the current prospective study where CTC evaluation and phenotypic analysis were performed at baseline, post‐1st cycle (3 weeks after baseline), post‐3rd cycle (9 weeks after baseline), and at primary resistance. Primary resistance [7] was defined as progression by RECIST criteria at first computed tomography (CT) evaluation.

Liquid biopsy samples were also obtained at post‐7th cycle (21 weeks after baseline), post‐10th cycle (30 weeks after baseline), post‐15th cycle (45 weeks after baseline), post‐18th cycle (54 weeks after baseline), and at secondary resistance [7] for patients who experienced an initial clinical benefit followed by the development of progression. Initial clinical benefit was determined as alive with complete response (CR) or partial response (PR) or with stable disease (SD) by RECIST at the first CT scan. Scheduled CT was performed every 9 weeks.

In the present study, all patients who were categorized as progressive disease (PD) by RECIST criteria had experienced disease progression at first CT evaluation of treatment (primary resistance). Furthermore, patients who had achieved an initial clinical benefit such as CR, PR, or SD were categorized as disease control (DC). Among 48 patients, all but one were evaluable for response according to RECIST criteria (Table 1). Twenty‐one (45%) experienced primary resistance, whereas 26 (56%) had DC. The median progression‐free survival (PFS) for PD and DC patients was 1.9 months (range: 0.8–2.1) and 9 months (range: 3.2–36.3), respectively, and the median overall survival (OS) was 7.7 months (range: 1.1–44.3) for PD and 17.5 months (range: 3.2–48.5) for DC patients.

**Table 1:** Clinical and pathological characteristics of the patient population. CR, Complete response; *n*, number of patients; n/a, not available; PD, progression disease; PR, partial response; SD, stable disease.

Age at diagnosis (years), *n* = 48
Median (range): 66 (40–82)
Sex, *n* = 48, *n* (%)
Male, 39 (81%)
Female, 9 (19%)
Histology, *n* = 48, *n* (%)
Adenocarcinoma, 35 (73%)
Squamous, 13 (27%)
Previous treatment (*n* = 48), *n* (%)
Platinum doublet chemotherapy, 48 (100%)
Immune checkpoint inhibitor (*n* = 48), *n* (%)
Anti‐PD1(pembrolizumab), 48 (100%)
Response according to RECIST, *n* = 48, *n* (%)
CR 0 (0%)
PR 2 (4%)
PD 21 (44%)
SD 24 (50%)
n/a 1 (2%)

### 2.2 Sample collection and cytospin preparation

Twenty milliliters of blood was obtained from each patient at each time point. To avoid blood contamination by epithelial cells from the skin, all blood samples obtained after the first 5 mL of blood were discarded. Peripheral blood mononuclear cells (PBMCs) were isolated with Ficoll–Hypaque density gradient (*d* = 1077 g·mol^−1^) centrifugation at 670 ***g*** for 30 min. PBMCs were washed three times with phosphate‐buffered saline solution (PBS) and centrifuged at 530 ***g*** for 10 min. Aliquots of 10^6^ cells were centrifuged at 700 ***g*** for 2 min on glass slides. Cytospins were dried up and stored at −80 °C. A total of 6 × 10^6^ PBMCs per patient were analyzed and the results are expressed as CTCs/6 × 10^6^ PBMCs as shown in Table S1.

### 2.3 Cell cultures and spiked cells

The lung cancer cell lines A549, H460, and H1975 were obtained from the American Type Culture Collection (Manassas, VA, USA). Cells were cultured in the appropriate medium supplemented with 10% fetal bovine serum (FBS) (GIBCO‐BRL) and 50 μg·mL^−1^ penicillin/streptomycin, in the appropriate conditions. Cell line cultures were screened for mycoplasma contamination using PCR and all experiments were performed during the logarithmic growth phase of cells. NSCLC cell lines were incubated with IFN‐γ (100 ng·mL^−1^) for 24 h, and cell lysates before and after IFN‐γ treatment were used for the evaluation of basal and induced levels of PD‐L1 protein by immunoblotting analysis. Cells were centrifuged on cytospins according to the procedure followed for patients' samples and cytospins of cell lines spiked into healthy volunteers' PBMCs (10^4^/250 000 PBMCs) were also prepared.

### 2.4 Immunoblotting analysis

Cell lysates and immunoblotting were prepared as previously described [20]. Briefly, 40 μg of cell lysates was used for PD‐L1 evaluation. Blots were incubated at 4 °C overnight, in blocking buffer with a rabbit antibody against PD‐L1 (E1L3N, Cell Signaling Technology Inc., Danvers, MA, USA) or a mouse antibody against GAPDH (SCBT Inc., Dallas, TX, USA). The expression levels of PD‐L1 in the NSCLC cell lines by western blot are shown in Fig. S1.

### 2.5 Immunostaining experiments

PBMCs' cytospins for each patient were triple stained with pancytokeratin, PD‐L1, and Ki67. Briefly, cytospin fixation and permeabilization were performed with ice‐cold acetone/methanol, 9/1 (V/V) for 20 min at RT, followed by incubation with blocking buffer (PBS/10% normal goat serum) for 60 min. Detection of cytokeratin‐positive cells was performed using the pancytokeratin mouse antibody, (AE1/AE3+5D3, Abcam, Cambridge, UK), 1 : 100 dilution overnight at 4 °C. This was followed by incubation with the FITC antibody, diluted 1 : 400 (Invitrogen, Thermo Fisher Scientific, Waltham, MA, USA) for 50 min. The cytomorphological criteria proposed by Meng and colleagues (i.e., high nuclear to cytoplasmic ratio and larger than white blood cells in size) were used to characterize a CK‐positive cell as a CTC [21]. To evaluate PD‐L1 expression, cells were stained with the PD‐L1 rabbit antibody (E1L3N, Cell Signaling Technology Inc.) diluted 1 : 100 for 1 h, followed by the secondary Alexa555 antibody (Invitrogen, Thermo Fisher Scientific) diluted 1 : 500 for 50 min. To identify Ki67 expression, cells were labeled with the rabbit Ki67‐conjugated with Alexa 647 antibody (Abcam) diluted 1 : 150 for 1 h. Finally, 4′,6‐diamidino‐2‐phenylindole (DAPI) reagent (Invitrogen, Thermo Fisher Scientific) was added to each sample for nuclear staining.

To evaluate the specificity of the antibodies, cytospins of cell lines spiked into healthy volunteers' PBMCs were used as positive and negative controls. Negative controls were prepared by omitting the corresponding primary antibody and adding the secondary IgG isotype antibody (Figs S2A,B and S3A). Moreover, cytospins consisting of H1975 cells spiked in PBMCs were stained along with patients' samples in order to standardize the staining protocol, the background from PD‐L1‐positive PBMCs, microscope settings, different antibodies' batch, during the whole period of the study. Cytospins were evaluated with a Leica microscope and the leica lasx software [22, 23].

Furthermore, in patients presenting high CTC numbers, PBMCs' cytospins were double stained with pancytokeratin and the common leukocyte antigen CD45. For double staining experiments, PBMC cytospins were incubated with the pancytokeratin mouse antibody diluted 1 : 100, overnight at 4 °C followed by the corresponding secondary FITC antibody for 50 min and the Alexafluor 647 anti‐human CD45 (HI30, BioLegend, San Diego, CA, USA) diluted 1 : 300 for 1 h. DAPI was added to each sample for nuclear staining. The specificity of the antibodies staining and a representative image of a CTC (CK^+^/CD45^−^) from a patient with high CTC number are shown in Fig. S4i–iv.

### 2.6 Semi‐quantitative analysis for PD‐L1 on CTCs

For the evaluation of PD‐L1 on CTCs of NSCLC patients, a semi‐quantitative analysis of PD‐L1 expression was performed, using three NSCLC cell lines (H1975, H460, and A549) expressing different levels of PD‐L1 protein as determined by western blot and triple immunofluorescence analysis (Figs S1 and S3B). The rabbit monoclonal antibody E1L3N was used to detect endogenous levels of total PD‐L1 protein. PD‐L1 expression was considered positive when localized in the cellular membrane, the cytoplasmic component, or both.

The Mean Fluorescence Intensity per pixel (MFI) of PD‐L1 expression for each cell was measured using the Leica Application Suite X (lasx) software (Leica Microsystems). The intensity of PD‐L1 protein was measured among PD‐L1 expressing cells detected in the positive control of H1975, H460, and A549 cells (Fig. S3B). Based on the mean intensity of the selected cell lines, we characterized CTCs as PD‐L1 high, medium, low, and negative, Fig. S5.

### 2.7 Statistical analysis

All statistical analyses were performed using graphpad prism 5 (GraphPad Software, San Diego, CA, USA) and ibm spss statistics, version 25 (IBM Corp., Armonk, NY, USA). Fisher's exact test and Wilcoxon matched‐pairs signed rank test were performed and Kaplan–Meier analysis was used to estimate survival curves; PFS was calculated from the day of the administration of the first cycle of pembrolizumab until the day of documentation of disease progression or death from any cause. Overall survival (OS) was calculated from the day of treatment initiation until the date of death from any cause. Cox regression analysis was performed to investigate the risk for progression and death. *P* values were calculated by two‐sided tests and were considered statistically significant at the 0.05 level.

## 3 Results

### 3.1 Quantification of PD‐L1 on cell lines and setting‐up the thresholds in CTCs of NSCLC patients using the lasx software

For this purpose, the Mean Fluorescence Intensity per pixel (MFI) of PD‐L1 expression for each cell expressing PD‐L1 was measured by LASX and the mean intensity of PD‐L1 staining from 300 cells in each cell line was used to set‐up the thresholds for classifying CTCs as high, medium, low, or negative (Fig. S4). The highest mean intensity was observed in H1975 cells (mean ± SD; 58 ± 16), a medium intensity was observed in H460 cells (39 ± 5.5) and the lowest intensity was observed in A549 cells (24 ± 5.3). Therefore, CTCs with MFI < 24 were considered negative and labeled as PD‐L1neg; if MFI was 25 ≤ MFI ≤ 38, cells were classified as low (PD‐L1low); if MFI was 39 ≤ MFI ≤ 57, cells were classified as medium (PD‐L1med); if MFI was ≥ 58 cells were characterized as high (PD‐L1high). The differential expression of PD‐L1 and Ki67 in CTCs, as determined by the triple immunofluorescence assay and quantified as described above, is shown in Fig. S6. Subsequently, CTCs were characterized as PD‐L1neg, low, medium, and high, and Ki67^+^ or Ki67^−^.

### 3.2 Evaluation of CTCs detected in longitudinal patients' samples

**Fig. 1:**
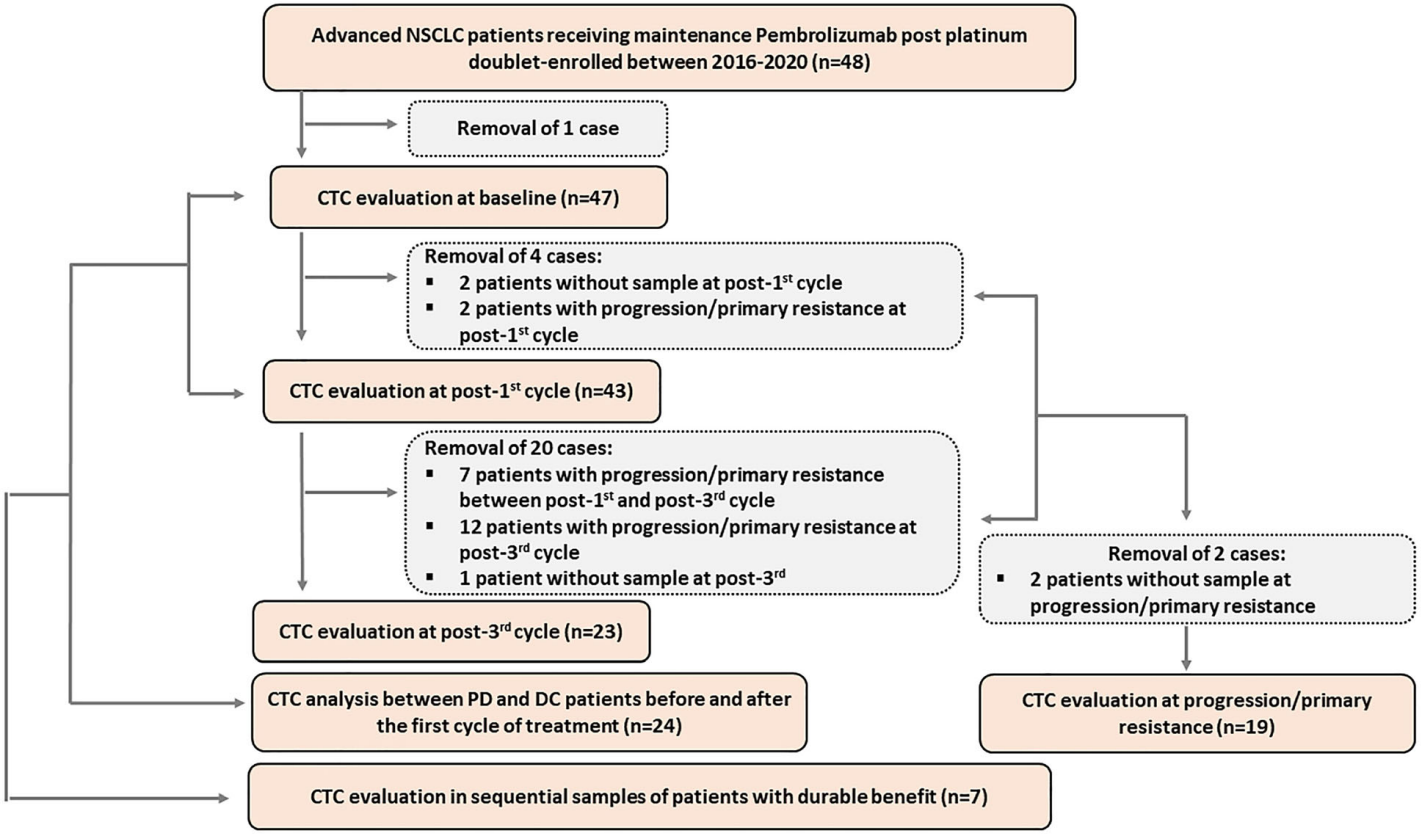
Flow chart showing the outline of circulating tumor cell (CTC) analysis in samples of patients treated with pembrolizumab (DC, disease control; *n*, number of patients; PD, progressive disease).

A total number of 48 advanced NSCLC patients were enrolled at the Bank of Cyprus Oncology Centre (BOCOC). One patient was excluded from the study because RECIST response was not available. A flow chart showing CTC analysis of patients' samples at baseline (*n* = 47), post‐first cycle (*n* = 43), post‐third cycle (*n* = 23), and progression/primary resistance (PMR), (*n* = 19) is depicted in Fig. 1. Patients' characteristics are shown in Table 1 and CTC evaluation at baseline, post‐1st, post‐3rd cycle, and primary resistance is depicted in Table S1.

#### 3.2.1 CTC evaluation before the initiation of treatment (baseline)

At baseline, CTCs were evaluated in 47 patients and detected in 44 (94%) patients (median CTC number: 3, range: 1–150), PD‐L1‐positive CTCs were detected in 38 (86%) patients (median % of PD‐L1‐positive CTCs: 98.5%, range: 0–100%). PD‐L1high, med, low, and negative CTCs were detected in 24 (55%), 24 (55%), 23 (52%), and 22 (50%) patients, respectively. The median % of the respective subpopulations were PD‐L1high: 2%, med: 4.5%, low: 8%, negative: 1.5% (range: 0–100%). Ki67^+^ CTCs were detected in 27 (61%) patients. The median % of Ki67^+^ CTCs were 20% (range: 0–100%).

#### 3.2.2 CTC evaluation after the first cycle (post‐first cycle)

At post‐1st cycle, CTCs were evaluated in 43 patients and detected in 36 patients (84%), (median CTC number: 4.5, range: 1–1022), PD‐L1‐positive CTCs were detected in 32 (89%) patients (median % of PD‐L1‐positive CTCs: 83%, range: 0–100%). PD‐L1high, med, low, and negative CTCs were detected in 17 (47%), 22 (61%), 19 (53%), and 21 (58%) patients, respectively. The median percentages of the respective subpopulations were PD‐L1high: 0%, med: 16%, low: 9.5%, negative: 17% (range: 0–100%). Ki67^+^ CTCs were detected in 23 (64%) patients. The median % of Ki67^+^ CTCs were 20% (range: 0–100%).

#### 3.2.3 CTC evaluation after the third cycle (post‐third cycle)

At post‐3rd cycle, CTCs were evaluated in 23 patients and detected in 17 (74%), (median CTC number: 2, range: 1–41), PD‐L1‐positive CTCs were detected in 13 patients (76%) (median % of PD‐L1‐positive CTCs: 100%, range: 0–100%). PD‐L1high, med, low and negative CTCs were detected in 4 (24%), 5 (29%), 9 (53%), and 7 (41%) patients, respectively. The median % of the respective subpopulations were PD‐L1high: 0%, med: 0%, low: 33%, negative: 0%, range: 0–100%. Ki67^+^ CTCs were detected in 6 (35%) patients (median % of Ki67^+^ CTCs: 0%, range: 0–100%).

#### 3.2.4 CTC evaluation at primary resistance (PMR)

For patients who experienced progression at first treatment evaluation (primary resistance), CTCs were examined in 19 patients and detected in 17 (89%) patients (median CTC number: 9, range: 1–1094), PD‐L1‐positive CTCs were detected in 14 (82%) patients (median % of PD‐L1‐positive CTCs: 74%, range: 0–100%), PD‐L1high, med, low, and negative CTCs were detected in 6 (35%), 8 (47%), 13 (76%), and 9 (53%) patients, respectively. The median % of the respective PD‐L1 subpopulations were PD‐L1high: 0%, med: 0%, low: 33%, negative: 16%, range: 0–100%. Ki67^+^ CTCs were detected in 14 (82%) patients (median % of Ki67^+^ CTCs: 42%, range: 0–100%). The details (CTC number) of each patient at the selected time points are shown in Table S1.

We subsequently, compared the CTC status (total CTC counts, the number, and percentage of PD‐L1 and Ki67 CTCs) between baseline and post‐first cycle, baseline, and post‐third as well as baseline and PMR. All comparisons were performed among CTC‐positive patients at both time points and analyzed by Wilcoxon‐matched pairs signed rank test. There were no significant differences in total CTCs, PD‐L1, and Ki67 subpopulations of patients between baseline and post‐first or baseline and post‐third cycle (data not shown). However, important changes in the PD‐L1 subpopulations were observed between baseline and primary resistance.

**Fig. 2:**
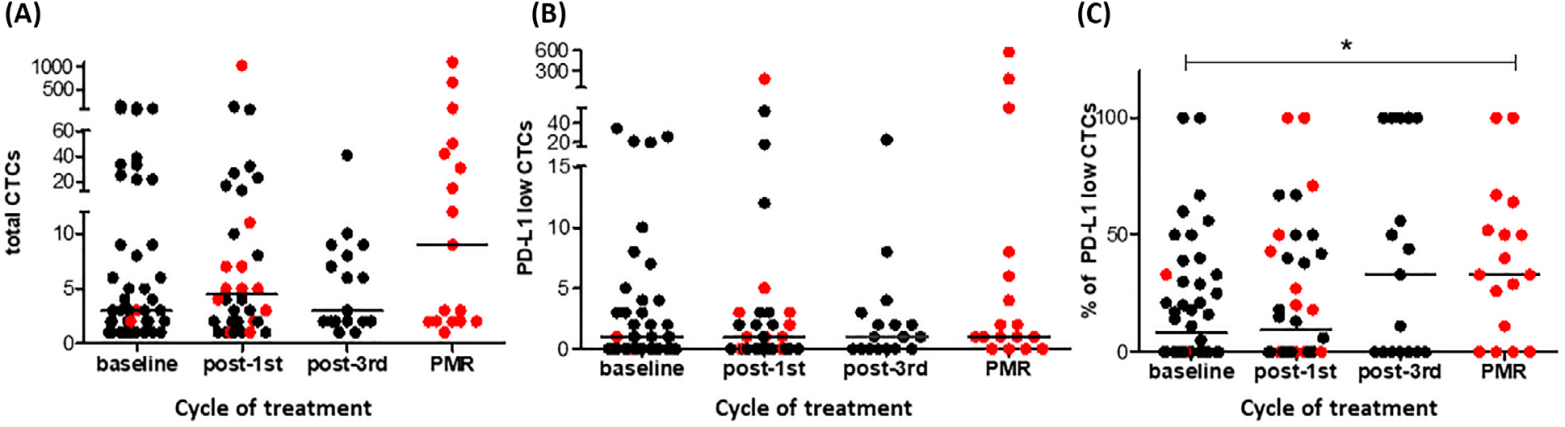
Longitudinal evaluation of the circulating tumor cells (CTCs) detected in patients at different timepoints of treatment; baseline (*n* = 44), post‐1st cycle (*n* = 36), post‐3rd (*n* = 17), and primary resistance (PMR) (*n* = 17). The total CTC number, PD‐L1low CTCs from each patient, and the PD‐L1low CTC rate at each evaluation time‐point are depicted in A, B, and C, respectively. Red dots display the patients who exhibited progression in the next cycle of treatment and are separately analyzed at PMR time point. Horizontal lines indicate mean values. *P < 0.05, *P*‐value was calculated by Wilcoxon‐matched pairs signed rank test.

Among CTC‐positive patients at both time‐points (*n* = 16), total CTCs were numerically increased; median number: 3 (range: 1–150) at baseline versus 10.5 (range: 1–1094) at PMR, *P* = 0.4. The number of PD‐L1low CTCs was subsequently raised; median number: 0.5 (range: 0–35) versus 1.5 (0–571), respectively, *P* = 0.1. Importantly, the percentage of PD‐L1low CTCs was significantly increased; median%: 2.5% (range: 0–60%) at baseline versus 36.5% (range: 0–100%) at PMR, *P* = 0.03. Fig. 2A–C show the longitudinal evaluation at baseline, post‐1st cycle, post‐3rd, and at PMR of the total CTC number, PD‐L1low CTCs, and the percentage or rate of PD‐L1low CTCs for each patient, respectively.

Furthermore, the number of PD‐L1high CTCs declined at PMR; median number: 1.5 (range: 0–49) at baseline versus 0 (range: 0–45) at PMR, *P* = 0.1, and the percentage or rate of PD‐L1high CTCs was significantly decreased; median%: 31.5% (range: 0%–100%) at baseline versus 0% (range: 0%–50%) at PMR, *P* = 0.008. No significant alterations were observed in the number and percentage of PD‐L1med, PD‐L1negative, and Ki67^+^ CTCs among CTC‐positive patients between the baseline and PMR.

Interestingly, different drifts of the PD‐L1 subpopulations were observed at primary resistance suggesting that changes in both PD‐L1high and PD‐L1low subpopulations may be involved.

### 3.3 CTC analysis between patients with primary resistance and disease control before and after the first cycle of treatment

To validate our results, we investigated CTC status between patients with primary resistance (PD patients) and DC, before and after the first cycle of treatment. Specifically, we examined changes in total CTCs, PD‐L1, and Ki67 subpopulations in PD and DC patients presenting CTCs ≥ 1 and CTCs > 1. CTC changes were classified as ‘increased’ and ‘decreased or not changed’. Specifically, if the CTC number increased by one or more cell after the first cycle, this was defined as an ‘increase’ in CTCs. Similarly, if the CTC number decreased by one or more cell after first cycle, this was defined as a ‘decrease’ in CTCs. However, significant changes were observed only among patients presenting CTCs > 1.

**Fig. 3:**
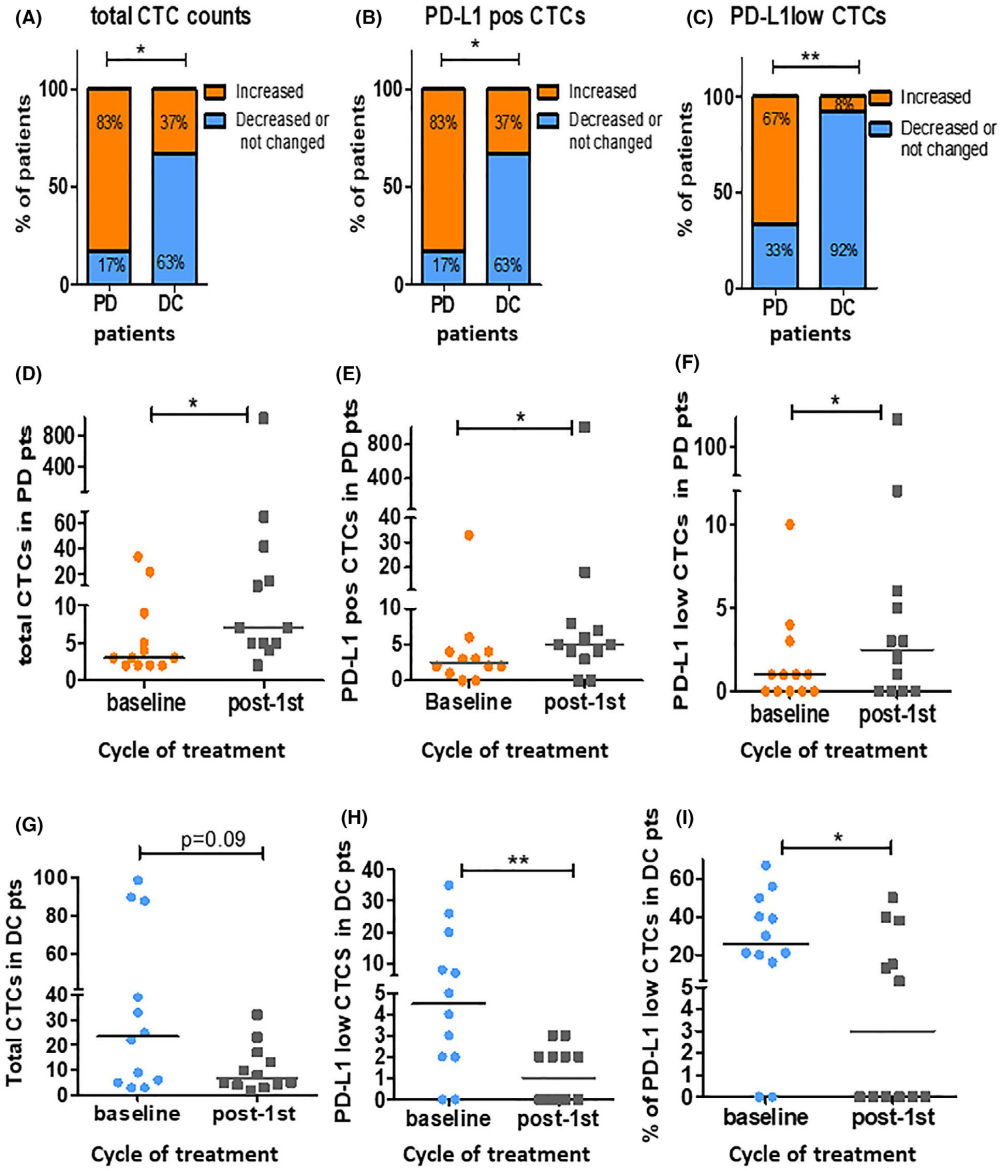
Changes in circulating tumor cell (CTC) status were compared between progression disease (PD) and disease control (DC) patients before and after the first cycle of treatment (*n* = 24). The increased versus decreased or not changed of (A) total CTC counts, (B) PD‐L1‐positive CTCs, and (C) PD‐L1 low CTCs were compared between PD and DC patients. The number of (D) total CTC counts, (E) PD‐L1‐positive, and (F) PD‐L1 low CTCs were significantly increased in PD patients at post‐1st cycle compared to baseline, whereas the number of (G) total CTCs, (H) PD‐L1low CTCs and (I) the % of PD‐L1low CTCs were decreased in DC patients compared to baseline, *P < 0.05, and **P < 0.01. (D–I) Horizontal lines indicate mean values. From (A) to (C), *P*‐values were calculated by Fisher's exact test and from (D) to (I) by Wilcoxon‐matched pairs signed rank test.

Twenty‐four patients were CTC positive at both time points and presenting CTCs > 1. Among them, 12 patients were classified as PD, while the rest as DC. After the first cycle of treatment, PD patients more frequently increased both total CTC counts and PD‐L1‐positive CTCs compared to DC patients: 83% (10/12) of PD patients versus 37% (4/12) of DC, *P* = 0.03 (Fig. 3A,B).

Interestingly, among the different PD‐L1 subpopulations, significant changes were observed only in the PD‐L1low CTC subset. Specifically, PD‐L1low CTC counts were more often increased in PD patients compared to DC: 67% (8/12) of PD patients versus 8% (1/12) of DC, *P* = 0.004, Fig. 3C.

Furthermore, the number of total CTCs, PD‐L1‐positive, and PD‐L1low CTCs were significantly raised in PD patients at post‐1st; median number of total CTCs: 3 (range: 2–34) at baseline versus 7 at post‐1st cycle (range: 2–1022), *P* = 0.01; median number of PD‐L1‐positive CTCs: 2.5 (range: 0–33) versus 5 (range: 0–998), respectively, *P* = 0.01; median number of PD‐L1 low CTCs:1 (range:0–10) versus 2.5 (range: 0–184), *P* = 0.02, Fig. 3D–F, respectively. The percentage of the PD‐L1‐positive CTCs was numerically increased; median%: 73% (range: 0–100%) at baseline versus 78% (range: 0–100%) at post‐1st, *P* = 0.7, and the percentage of PD‐L1low CTCs was numerically increased; median%: 14.5% (range: 0–60%) at baseline versus 19% (range: 0–71%) at post‐1st cycle, *P* = 0.6.

On the other hand, DC patients more frequently decreased or not changed PD‐L1low CTC counts compared to patients with PD: 92% (11/12) of DC versus 33% (3/12) of PD, *P* = 0.004, Fig. 3C. Total CTCs, PD‐L1low CTCs, and the percentage of PD‐L1low CTCs were also decreased in DC patients; median number of total CTCs:23.5 (range: 3–99) at baseline versus 6.5 (range: 2–32) at post‐1st, *P* = 0.09; median number of PD‐L1low CTCs: 4.5 (range: 0–35) versus 2 (range: 0–3), respectively, *P* = 0.007; median % of PD‐L1low CTCs: 25% (range: 0–67%) versus 3% (range: 0–50%) *P* = 0.049, Fig. 3G–I, respectively.

However, there were no significant differences before and after the first cycle, in the PD‐L1high, PD‐L1med, PD‐L1neg, and Ki67^+^ subpopulations between PD and DC patients (Fig. S7).

### 3.4 Association of the CTC status with the clinical outcome

**Fig. 4:**
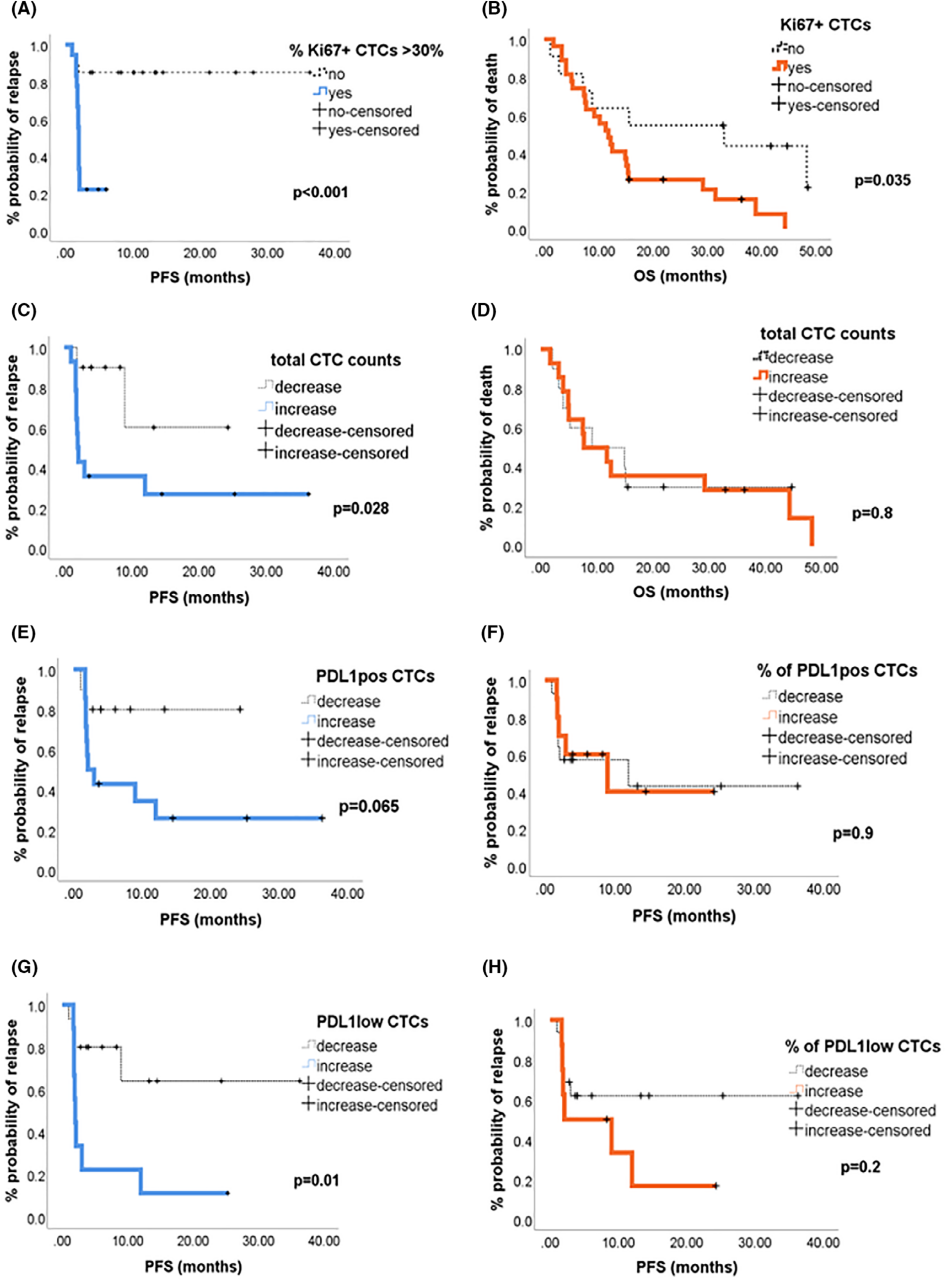
Prognostic relevance according to % of Ki67^+^ circulating tumor cells (CTCs) and Ki67^+^ CTCs in PD‐L1^+^ patients (*n* = 38) prior to treatment, Kaplan–Meier curves for progression‐free survival (PFS) (A) and overall survival (OS) (B), respectively. Prognostic relevance according to CTC status change in patients presenting CTCs > 1 between baseline and post‐first cycle (*n* = 24). Kaplan–Meier curves for PFS (C) and OS (D) according to the change (decrease or increase) of total CTC counts. PFS curves for decrease or increase in the PD‐L1‐positive CTCs (E), percentage (%) of PD‐L1positive CTCs (F), PD‐L1low CTCs (G), and percentage (%) of PD‐L1low CTCs (H), *P*‐values were calculated by the log‐rank test.

We further investigated whether the differential expression of PD‐L1 and Ki67 as well as the CTC changes during pembrolizumab treatment were associated with the clinical outcome. We showed that before treatment the majority of patients (86%), were PD‐L1^+^. Subsequently, we investigated whether the presence of Ki67^+^ CTCs could affect patient's outcome. We found that PD‐L1^+^ patients harboring Ki67^+^ CTCs had a trend for shorter PFS compared to those not presenting (*P* = 0.083). Moreover, PD‐L1^+^ patients presenting a high Ki67 index (% Ki67^+^ CTCs > 30%) prior to treatment showed a significantly shorter PFS compared to those with a low (% Ki67^+^ CTCs ≤ 30%; median PFS: 2 months versus not reached, respectively, *P* < 0.001, Fig. 4A). Cox regression analysis revealed an increased risk of relapse for patients with high Ki67 index; hazard ratio (HR): 7.2, *P* = 0.002. In addition, PD‐L1^+^ patients harboring Ki67^+^ CTCs had a shorter OS compared to those not presenting (median OS: 11.8 months vs. 33.1 months, *P* = 0.035; HR: 2.6, *P* = 0.042, Fig. 4B).

Furthermore, among patients displaying CTCs > 1, those with increased CTC counts at post‐1st cycle had significantly shorter PFS compared to those with decreased CTC counts (median PFS: 2 months vs. not reached, *P* = 0.028; HR: 4.6, *P* = 0.04, Fig. 4C). There was no significant difference in the OS between patients with increased CTC counts and those with decreased counts, *P* = 0.8, Fig. 4D.

Interestingly, patients presenting a decrease in PD‐L1‐positive CTCs had a trend for longer PFS compared to those with increased PD‐L1‐positive CTCs (*P* = 0.065), Fig. 4E. There was no significant difference in the PFS between patients presenting a decrease in PD‐L1‐positive CTC percentage and in those with increased PD‐L1‐positive CTC percentage, *P* = 0.9, Fig. 4F.

Finally, patients showing a decrease in PD‐L1low CTC counts after the first cycle had significantly longer PFS compared to those with increased PD‐L1low CTC counts (median PFS: not reached vs. 2 months, *P* = 0.01; HR: 4.2, *P* = 0.02), Fig. 4G. However, no significant difference was observed in the PFS between patients presenting decrease in PD‐L1low CTC percentage and in those with increased PD‐L1low CTC percentage, *P* = 0.2, Fig. 4H.

### 3.5 Evaluation of CTCs in sequential follow‐up samples of patients with long‐term benefit from treatment

To investigate whether changes in CTC status could be associated with a durable benefit from pembrolizumab treatment, total CTCs, PD‐L1, and Ki67 subpopulations were evaluated in serial samples of patients with a long PFS period.

**Fig. 5:**
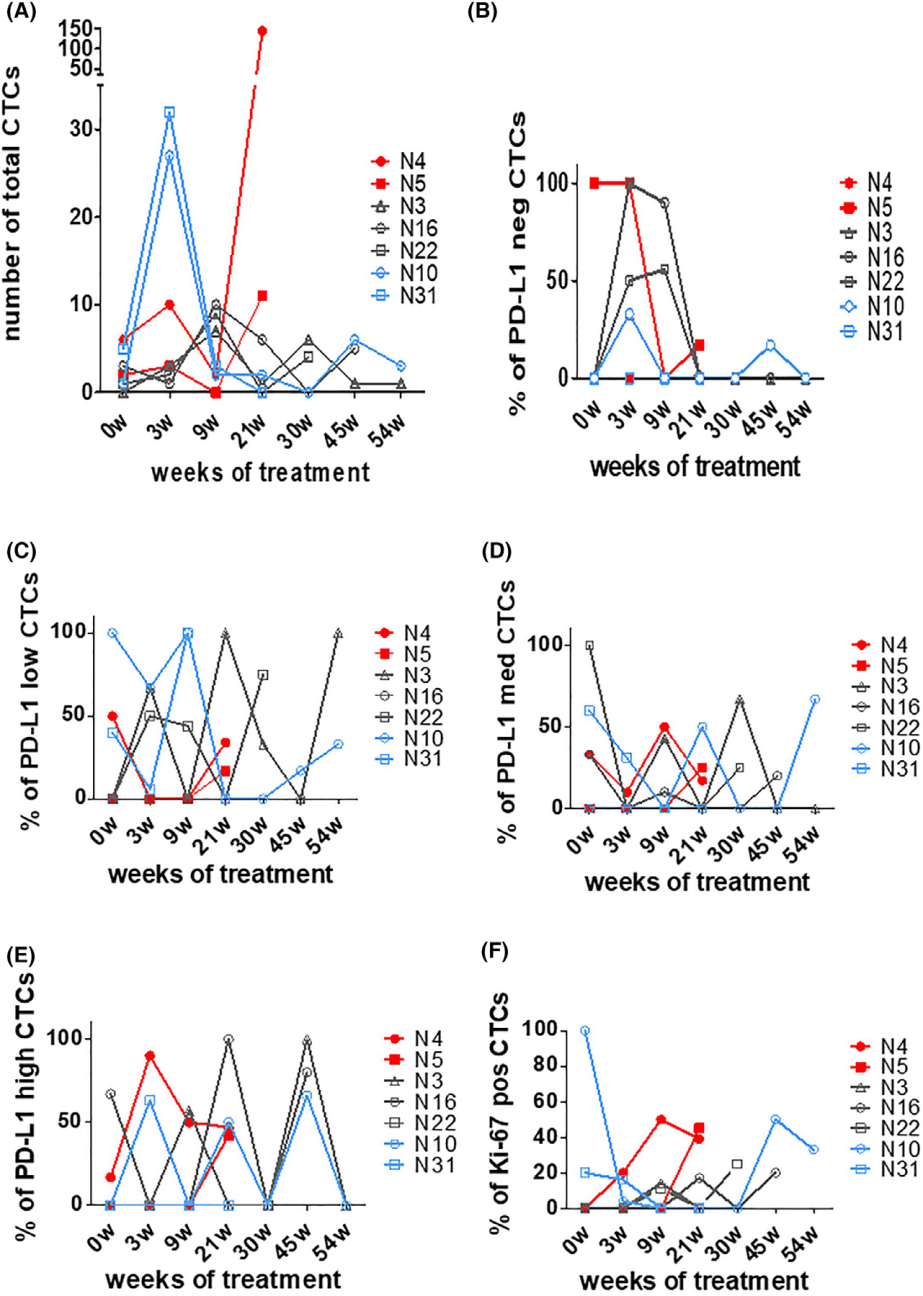
Changes in circulating tumor cell (CTC) status in serial samples of patients treated for long‐term with pembrolizumab (A) number of total CTCs, (B) percentage (%) of PD‐L1negative CTCs, (C) % of PD‐L1low, (D) % of PD‐L1med, (E) % of PD‐L1high and (F) % of Ki67positive CTCs. Patients were categorized into three groups. Blue lines show the first group (N10 and N31 patients); red shows the second group (N4 and N5 patients) and gray shows the third group (N3, N16, and N23 patients).

However, the number of CTCs identified in majority of patients' samples was very low and CTCs were not detected in all evaluation time points. Herein, we present serial samples of seven patients in whom CTCs were harvested at most of the sequential time points (0, 3, 9, 21, 30, 45, and 54 weeks). The median PFS and OS of these patients were 13.3 and 44.7 months, respectively. Figure 5A depicts the total CTC number detected in the sequential follow‐up samples of patients: N3, N4, N5, N10, N16, N22, and N31.

Five of the above patients had achieved a stable disease at first CT evaluation, before experienced a subsequent disease progression or secondary resistance, whereas only two patients (N10 and N31) had shown partial response among all the patients who participated in the study. Patients N4 and N5 experienced disease progression at 17 and 24 weeks, while patients N22, N16, and N3 experienced progression, at 40, 52, and 76 weeks, respectively, after treatment initiation. Furthermore, patients N10 and N31 have not experienced any disease progression until recently.

Interestingly, we observed that in patients with partial response (N10 and N31), CTC counts were immediately increased at week 3, whereas the PD‐L1low CTC rates were decreased (Fig. 5A,C, blue lines). Moreover, fewer CTCs were detected in their following serial samples (Fig. 5A). The percentage of PD‐L1high CTCs was increased at week 3 and at week 21 in N10 and N31 patients, respectively (Fig. 5E). PD‐L1neg CTCs were identified at week 3 and at week 45 for patient N10, whereas PD‐L1neg CTCs were not detected in patient N31 for the whole examination period (Fig. 5B). Interestingly, the Ki67 CTC index remained low (≤ 30%) during the whole evaluation period for these patients (Fig. 5F).

On the other hand, in N4 and N5 patients, total CTC counts were peaked at week 21 (point of progression) and PD‐L1low CTC rates were also increased at this time point (Fig. 5A,C, red lines). Interestingly, the Ki67 CTC index was also high on disease progression (> 30%) (Fig. 5F).

In patients N3, N16, and N22 who continued to benefit from pembrolizumab treatment for more than 21 weeks, fewer CTC counts were detected through the whole examination period (Fig. 5A, gray lines) and PD‐L1neg CTCs were detected at weeks 3 and 9. PD‐L1low or PD‐L1high CTC rates decreased or increased at each evaluation time point until week 54 (Fig. 5C,E) and the Ki67 CTC index also retained low ≤30% (Fig. 5F).

## 4 Discussion

In the present study, we examined the differential expression of PD‐L1 and the proliferation marker Ki67 on CTCs in longitudinal samples of NSCLC patients treated with pembrolizumab. We validated our findings between patients with primary resistance and disease control before and after the first cycle of treatment. Furthermore, CTC changes were investigated in sequential samples of patients who obtained a long‐term benefit from treatment.

We initially hypothesized that CTC evaluation in an early phase of pembrolizumab treatment, could reveal significant changes in PD‐L1 subpopulations which might be associated with resistance or response to immunotherapy. Interestingly in our cohort of patients, all cases who were characterized as progressive disease (PD) by RECIST had experienced primary resistance. Subsequently, the CTC status was examined at baseline, post‐first, post‐third cycle, and the point of primary resistance.

In the current study, using the ficoll density gradient centrifugation, an antigen‐independent method, and evaluating 6 × 10^6^ PBMCs per patient, high CTC detection rates were observed. Several studies using different CTC isolation and detection methods have shown varied CTC detection rates, ranging from 50 to 100%. Specifically, in NSCLC, fewer CTCs can be detected with antigen‐dependent methods compared to antigen‐independent methods [24]. Krebs et al. [25] utilizing the latter method (ISET) observed CTCs in 80% of stage III–IV NSCLC patients, whereas with the use of an antigen‐dependent method (CellSearch®) CTCs were identified in only 22.5% of the same patients. Recently, Janning et al., using the Parsortix system, another label‐independent method showed that CTCs ≥1, were detected in significantly more NSCLC patients compared to the CellSearch (60.8% vs. 31.9%) [26, 27]. While both ISET and Parsortix CTC enrichment methods use the whole blood without any previous process, the ficoll enrichment utilizes only the PBMC fraction which causes the loss of some CTCs. Moreover, CTCs will be detected in a part of PBMCs. The high CTC detection rate we observed in this investigation can be explained by the antigen‐independent method we used as well as by the evaluation of a large number of PBMCs per patient (6 million PBMCs).

Another interesting approach in our investigation was that CTCs were classified into four PD‐L1 subcategories (high, medium, low, and negative). In general, cancer cells expressing PD‐L1 bind to PD‐1 receptor on T cells and effectively inhibit T‐cell proliferation. However, it has been reported that engagement of different levels (low, intermediate, and high) of both PD‐1 receptor and its ligand PD‐L1 can modulate the strength of PD‐1 signaling and affect a variety of T‐cell functions, such as cytokines and IFN‐γ production, TNFα, and IL‐2 derivation as well as T‐cell expansion [28]. Consequently, the evaluation of the different levels of PD‐L1 expression on CTCs could uncover which of the PD‐L1 subpopulations are actively involved in primary resistance or response to pembrolizumab treatment.

In the present study, no significant changes were found in the total CTC counts and PD‐L1 subpopulations (negative, low, medium, and high) between the baseline and post‐1st (3 weeks after treatment initiation) as well as between baseline and post‐3rd cycle (9 weeks after treatment). Our results are in line with a recent study of Ikeda et al. [29] who reported no significant changes in total CTC number after 4, 8, and 12 weeks of the beginning of Nivolumab treatment (another anti‐PD‐1 antibody). However, the authors reported a significant reduction in the rate of PD‐L1‐positive CTCs after 8 weeks of treatment.

Interestingly, we found a significant increase in the PD‐L1low CTC rate at primary resistance compared to that before treatment initiation (Fig. 2C), suggesting that enrichment of the PD‐L1low subpopulation may be associated with resistance to pembrolizumab. Previously, Wei et al. [28], demonstrated that a subtle amount of PD‐L1 is sufficient to mediate suppression of T‐cell proliferation. Therefore, we supposed that CTCs expressing PD‐L1low levels could effectively inhibit T‐cell expansion and suppress an immune response.

PD‐L1 is a dynamic and inducible biomarker that can be induced or maintained by many cytokines of which interferon‐γ (IFNγ) is the most potent. Activated T cells or innate immune cells can release IFNs and stimulate PD‐L1 expression. Moreover, the interaction between tumor‐infiltrating T cells, IFNγ signaling genes, and PD‐L1 expression suggests that activated T cells contribute to high levels of PD‐L1. Subsequently, we can assume that at the time of primary resistance due to the absence of activated T cells, no induction of PD‐L1 can be achieved. Therefore, it is logical to expect a decline in the PD‐L1 high CTC rate in patients with primary resistance. In support of our findings, Herbst et al., demonstrated a lack of PD‐L1 upregulation by either tumor cells or tumor‐infiltrating cells in tumor biopsies of early progressing NSCLC patients treated with ICI [5].

Immune profile analysis of cancer cells categorized tumors into ‘inflamed’ and ‘non‐inflamed’ types. Recent clinical studies in patients with melanoma cancer suggest that the ‘inflamed’ but not the ‘non‐inflamed’ tumors are associated with response to PD‐1 pathway blockade.

Interestingly, PD‐L1low expression in cancer and tumor‐infiltrating cells has been associated with ‘non‐inflamed tumors’ that may express high EMT and stem‐like features and show no‐response to ICIs treatment [30]. Therefore, it can reasonably be expected that patients with increased PD‐L1low CTCs may have more resistance to immunotherapy and subsequently present a poor clinical outcome.

On the other hand, it has been proposed that patients with ‘inflamed tumors’ display high levels of PD‐L1 expression on cancer and tumor‐infiltrating cells, harbor activated T cells and they are responsive to PD‐1 pathway blockade. Consequently, it is logical to assume that these patients will be able to reduce PD‐L1low CTCs and may have a better clinical outcome.

To validate our results, we further investigated CTC changes between patients with primary resistance (PD patients) and DC, before and after the first cycle of treatment. As PD‐L1 is an inducible dynamic factor that can change over time and treatment might affect its expression, it has been suggested that evaluation of PD‐L1 expression should be performed as close as possible to the initiation of treatment [31]. Therefore, changes in total CTCs, PD‐L1, and Ki67 subpopulations, between PD and DC patients were examined before and after the first cycle of pembrolizumab.

Interestingly, after the first cycle, total CTC counts, PD‐L1‐positive and PD‐L1low CTCs more often increased in PD patients compared to DC (Fig. 3A–C). Our results are in agreement with the study of Janning et al [26], who showed in a small cohort of NSCLC patients treated with PD‐1/PD‐L1 inhibitors that increase in PD‐L1‐positive CTCs was associated with resistance, whereas a decline in PD‐L1‐positive cells was related to response to treatment. However, we showed for the first time that a specific subpopulation of PD‐L1‐positive cells, the PD‐L1low CTC subset seems to be involved in immunotherapy resistance. Furthermore, Dhar et al. [32], quantified PD‐L1 expression levels in CTCs and tumor tissue in patients treated with ICIs and found that the majority of PD‐L1‐positive cells in both CTCs and tumor specimens had low PD‐L1 expression. Subsequently, it is more likely an elevation of PD‐L1‐positive cells to be associated with an increase in PD‐L1low CTCs rather than with other PD‐L1 subpopulations.

Another interesting finding in the present study was that PD‐L1low CTCs were decreased in almost all DC patients (Fig. 3C). This is the first study in a homogenous group of NSCLC patients treated with ICIs showing that a decrease in PD‐L1low CTCs is associated with a disease control state (partial response or stable disease).

On the other hand, it has been supported that CTC detection rates as well as the harvested CTC subpopulations may be dependent on the methodology used for CTC collection [33]. In this way, in a study including 35 patients with 11 different gastrointestinal tumors [34], the authors isolated CTCs with an EpCAM‐positive enrichment method, quantified PD‐L1 levels on CTCs, and only examined the distribution of PD‐L1high CTCs. They found that PD‐L1high CTC counts were increased in patients who experienced disease progression while they were decreased in patients with disease control (CR, PR, SD). However, this discrepancy could be related to the different methods used for CTC isolation, the PD‐L1 antibodies, the inconformity in treatment cycles, the short follow‐up time as well as the tumor type or histological subtype [35]. This aspect is further supported by a recent study in NSCLC patients treated with pembrolizumab, in which the authors identified PD‐L1positive CTCs in 20% of patients' samples, using the CellSearch platform, whereas PD‐L1positive CTCs were detected in 100% of the same patients using the Parsortix system [23]. It was suggested that the Parsortix system could allow the detection of CTCs with a more mesenchymal phenotype and subsequently a higher percentage of PD‐L1‐positive CTCs was detected.

Another important point of our study was that PFS was significantly longer in patients with decreased total CTC counts after the first cycle, compared to those with increased CTCs. This is consistent with previous studies showing that a decline in CTCs after chemotherapy [36, 37] or ICI treatment [38] is associated with a favorable response to treatment and improved survival, whereas the increase in CTCs may represent the loss of therapeutic benefit [39].

Interestingly, in another study, the authors reported that the presence of PD‐L1‐positive CTCs ≥ 1% of total cells in NSCLC patients before ICI treatment [16, 17] was associated with a bad prognosis and short survival. In the current investigation, we found that patients with reduced number of PD‐L1‐positive CTCs after first cycle showed a trend for longer PFS, but most importantly patients with decreased PD‐L1low CTC counts had a significantly longer PFS.

Herein, we showed for the first time, that changes in the number of PD‐L1low CTCs at an early evaluation time‐point of pembrolizumab such as 3 weeks after the beginning of immunotherapy, may have a predictive value for the treatment efficacy. This finding is further supported by our previous results that patients with disease control state and subsequently an active immune system can effectively reduce PD‐L1low CTCs. However, we found that decrease in the PD‐L1positive or PD‐L1low CTC rates was not associated with significant changes in the PFS of patients. This result could be explained by the changes in both PD‐L1negative and PD‐L1positive CTCs during treatment and by the fact that the rate of PD‐L1positive CTCs is dependent from the total number of CTCs detected [40].

In order to investigate whether CTC changes were predictive for long‐term efficacy of treatment, sequential follow‐up samples of patients with a durable clinical benefit were examined. Our results revealed that partial responders (N10 and N31), who had a prolonged PFS and OS period (PFS: 27.9 and 36.3 months and OS: 48.1 and 36.3 months, respectively), presented a rapid increase in CTC number just after the first cycle of treatment (3 weeks after the beginning of treatment), while the total CTC counts declined in the following treatment cycles (Fig. 5A). In line with our results, an immediate increase in CTC number was depicted in partial responders of NSCLC patients treated with nivolumab (4 weeks after treatment initiation), suggesting that an early increase in CTC counts was indicative of response and long‐term benefit from ICI treatment [29].

Accumulating evidence has also demonstrated that CTCs can mobilize from the tumor due to tissue disruption in an acute response to cancer treatment [41] and isolated CTCs have been detected as viable, Ki67^+^ cells or CTC clusters (2–50 cells) with increased metastatic potential [42].

Previous studies in NSCLC have shown that patients whose tumors presented a high Ki67 labeling index (> 30%) showed a poor prognosis, whereas patients with a low Ki67 index (≤ 30%) on tumor specimens, had a better clinical outcome [43, 44]. In this investigation, the percentage of Ki67^+^ CTCs had no significant changes in the early phase of treatment (post‐first, post‐third cycle, and primary resistance) compared to baseline. However, we demonstrated for the first time that PD‐L1^+^ patients presenting a low Ki67 CTC index (≤ 30%) prior to pembrolizumab, had a significantly longer PFS compared to those with a high Ki67 (> 30%), and PD‐L1^+^ patients harboring Ki67^+^ CTCs had a significantly lower OS compared to those not presenting. Our results suggest that Ki67 may have a predictive and prognostic role in PD‐L1^+^ metastatic NSCLC patients prior to immunotherapy. This is further supported by the observation that PD‐L1^+^ patients (N10, N31, N3, N16, and N22) who had a prolonged PFS period (PFS > 21 weeks), displayed a low Ki67 CTC index during treatment (Fig. 5F, blue and gray lines), whereas a high Ki67 was depicted in patients with disease progression at 21 weeks after pembrolizumab initiation.

Another interesting observation was that PD‐L1low CTC rates of patients who continued to benefit from pembrolizumab were increased at a later phase of treatment (Fig. 5C) and this was not associated with resistance to PD‐1 blockade. This is in contrast with that observed in the early time points. However, our results are consistent with the study of Ikeda et al [29], in which the authors reported that elevation of the PD‐L1 positivity rates during nivolumab treatment was a predictor of a long‐term efficacy of treatment rather than resistance.

Our results suggest that besides the evaluation of PD‐L1 expression by tumor biopsy, the differential expression of PD‐L1 and Ki67 on CTCs could provide further predictive information for NSCLC patients treated with ICIs. Nevertheless, our results and their interpretation, are subject to limitations. One limitation comprises the low CTC number detected in some patients and a further one, that significant changes between PD and DC patients were observed in patients presenting CTC number > 1. In addition, CTC detection was exclusively based on CK expression, eliminating the detection of mesenchymal CTCs. Our limitation of not using the hematopoietic marker CD45 in the antibodies panel, reflects the technical restriction of triple immunofluorescence assay in using up to three markers. Notwithstanding this, we confirmed the presence of CK‐positive/CD45‐negative cells, in patients presenting high CTC numbers.

To verify the significance of the PD‐L1low subpopulation as a predictive factor, our hypothesis should be validated in a larger homogenous cohort of patients including different PD‐L1 antibodies and CTC enrichment methods. Moreover, it should be investigated whether changes in PD‐L1 expression on CTCs are related to changes in tumor biopsies. Although, it is not feasible to obtain serial biopsies due to the invasiveness of the procedure, the examination of tumor tissue at baseline and on disease progression is essential to be investigated for this issue.

## 5 Conclusions

To summarize, this study investigates whether the differential expression of PD‐L1 and Ki67 on CTCs can provide further predictive information in advanced NSCLC patients treated with pembrolizumab. The quantification of PD‐L1 expression levels (high, medium, low, and negative) on CTCs and the evaluation of PD‐L1 subpopulations at different timepoints (baseline, post‐1st, post‐3rd, and primary resistance) revealed that changes in PD‐L1low subpopulation at an early phase of treatment, are importantly related to disease control or resistance to pembrolizumab immunotherapy. Furthermore, the evaluation of Ki67 prior to immunotherapy and in sequential follow‐up samples of patients treated with pembrolizumab, can possibly have a predictive and/or prognostic value for PD‐L1^+^ patients.

Finally, our findings suggest that monitoring the PD‐L1 and Ki67 expression in CTCs of NSCLC patients treated with pembrolizumab, might improve the selection of those patients that would potentially benefit from this therapy, reduce the medical cost, tailor patient's treatment, and improve the quality of patient's life.

## Conflict of interest

HC declares research institutional funding from MSD, and also Advisory Board participation with MSD, Novartis, Pfizer, Ipsen, with all fees collected by his institution. HC also declares travel expenses in relation to Oncology meetings covered by MSD, Roche, and Pfizer in the last three (3) years. All other contributors declare no conflict of interest.

## Author contributions

MS participated in study design and coordination, performed PBMCs isolation, cell cultures, and CTCs characterization, immunofluorescence, analyzed the results, and drafted the manuscript. CMN participated in laboratory work and in the preparation of the manuscript. PV was involved in data analysis. IS participated in data analysis. GG performed immunostaining experiments. CD co‐supervised the work and was involved in the preparation of the manuscript. AIC coordinated and supervised the study and was involved in the preparation of the manuscript. HC designed, coordinated, and supervised the study and was involved in data analysis and interpretation.

### Peer review

The peer review history for this article is available at https://publons.com/publon/10.1002/1878‐0261.13317.

## Supporting information


**Fig. S1.** PD‐L1 expression in NSCLC cell lines before and after induction with IFN‐γ by western blot. Representative western blot of three independent experiments. Total lysate (40μg) of NSCLC cell lines was electrophoresed by SDS‐PAGE (7.5% gel) and immunoblotted for total PD‐L1. Western blot showing strong baseline expression of PD‐L1 in H1975 NSCLC cells, medium in H460 and low expression in A549 cells. Treatment with 100 ng/ml interferon‐γ (IFN‐γ) for 24h upregulated PD‐L1 expression. The rabbit monoclonal antibody E1L3N was used to detect endogenous levels of total PD‐L1 protein and GAPDH served as a loading control.

**Fig. S2.** Expression of CK, PD‐L1 and Ki67 on H1975 (A) and H460 cells (B) spiked in peripheral blood mononuclear cells (PBMCs), by triple immunofluorescence staining. Cytospins of NSCLC cell lines spiked in PBMCs were used as positive and negative controls to evaluate the specificity of the antibodies used for the immunofluorescence staining. Cells were triple stained with pancytokeratin (CK) mouse antibody/secondary anti‐mouse FITC (green), anti‐PD‐L1 rabbit/ secondary anti‐rabbit Alexa Fluor 555 (red) and anti‐Ki67 rabbit 647‐conjugated antibody (grey). Cell nuclei were stained with DAPI (blue). The positive nuclear dotted staining (grey) was evaluated for Ki67 staining. Images were obtained using LASX, (x40).

**Fig. S3.** Expression of CK, PD‐L1 and Ki67 on A549 cells spiked in peripheral blood mononuclear cells (PBMCs) (A) and on NSCLC cell lines (B) by triple immunofluorescence staining. Cytospins of A549 cells spiked in PBMCs were used as positive and negative controls to examine the specificity of the antibodies used, whereas cytospins of H1975, A549 and H460 cells were used for the semi‐quantitative analysis of PD‐L1 expression. Cells were triple stained with pancytokeratin (CK) mouse antibody/secondary anti‐mouse FITC (green), anti‐PD‐L1 rabbit/ secondary anti‐rabbit Alexa Fluor 555 (red) and anti‐Ki67 rabbit 647‐conjugated antibody (grey). Cell nuclei were stained with DAPI (blue). The positive nuclear dotted staining (grey) was evaluated for Ki67 staining. Images were obtained using LASX, (x40).

**Fig. S4.** Double immunofluorescence staining with CK and CD45 antibodies on H460 cells spiked in peripheral blood mononuclear cells (PBMCs) and on PBMCs of NSCLC patients with high circulating tumor cell (CTC) number. (i–iii) Cytospins of H460 cells spiked in PBMCs were used as positive and negative controls to examine the specificity of CK and CD45 antibodies. Cells were double stained with pancytokeratin (CK) mouse antibody/secondary anti‐mouse FITC (green) and anti‐CD45, Alexafluor‐647 conjugated (grey). (iv) Representative image of a CK+/ CD45‐ cell among PBMCs from a patient with high CTC number. Images were obtained using LASX, (x40).

**Fig. S5.** Setting‐up the thresholds in circulating tumor cells based on three NSCLC cell lines with different PD‐L1 expression levels. The mean fluorescence intensity per pixel (MFI) of PD‐L1 expression in A549, H460 and H1975 cell lines was determined by imaging and analysis system LASX (Leica, microsystems). The mean intensity (m) of PD‐L1 staining in each cell line is used to define the thresholds for semi‐quantification and it was significantly different among the cell lines tested (*P<0.0001). The highest mean intensity was observed in H1975 cells (mean ± SD; 58 ± 16), a medium intensity of PD‐L1 was observed in H460 cells (39 ± 5.5) and the lowest intensity of PD‐L1 staining was observed in A549 cells (24 ± 5.3).

**Fig. S6.** Expression of CK, PD‐L1 and Ki67 on circulating tumor cells (CTCs) of NSCLC patients by triple immunofluorescence assay. Representative images of phenotypically different CTC subpopulations. Different scenarios of PD‐L1 and Ki67 differential expression in CTCs are shown in panels: (a) PD‐L1high and Ki67+ CTC, (b) PD‐L1high and Ki67‐ CTC among peripheral blood mononuclear cells (PBMCs), (c) two PD‐L1med and Ki67+ CTCs among PBMCs, (d) PD‐L1med and Ki67‐ CTC among PBMCs, (e) PD‐L1low and Ki67+ CTCs, (f) PD‐L1low and Ki67‐ CTC beside PBMCs, (g) PD‐L1neg and Ki67+ CTC among PBMCs and (h) three PD‐L1neg and Ki67‐ CTCs. Cell nuclei were stained with DAPI (blue), Ki67+ CTCs are shown with grey. Images were obtained using LASX, a Leica imaging and analysis system (x63).

**Fig. S7.** Changes in circulating tumor cell (CTC) status before and after first cycle, according to PD‐L1high, PD‐L1med, PD‐L1neg and Ki67+ CTCs between progression disease (PD) and disease control (DC) patients. The increased vs decreased or not changed of each CTC status was compared between PD and DC patients. No significant differences were observed in (a) PD‐L1high (b) PD‐L1med, (c) PD‐L1neg and (d) Ki67+ CTCs between PD and DC patients. *P*‐value was calculated by Fisher's exact test.

**Table S1.** Circulating tumor cell (CTC) enumeration and characterization according to PD‐L1 and Ki67 in 47 NSCLC patients during pembrolizumab treatment. CTC evaluation according to PD‐L1 and Ki67 in NSCLC patients: (i) at baseline, n=47 patients (ii) post‐first cycle, n=43 (iii) post‐third cycle, n=23 and (iv) at primary resistance, n=19 patients with disease progression at first evaluation of treatment (n/a: not applicable, n/s: no sample, “‐”: no disease progression at first evaluation). Patient's response according to RECIST criteria (PR: partial response, SD: stable disease and PD: progression disease). Results are expressed as CTCs/6x10^6^ PBMCs.

Appendix S1

## Data Availability

All data generated or analyzed during this study are included in the published article and its supporting information files.
