# Supplementary figures and images for: Dynamic monitoring of PD‐L1 and Ki67 in circulating tumor cells of metastatic non‐small cell lung cancer patients treated with pembrolizumab
Source: Mol Oncol. 2022 Dec 16;17(5):792–809. doi: 10.1002/1878-0261.13317 (PMC10158784; doi:10.1002/1878-0261.13317)

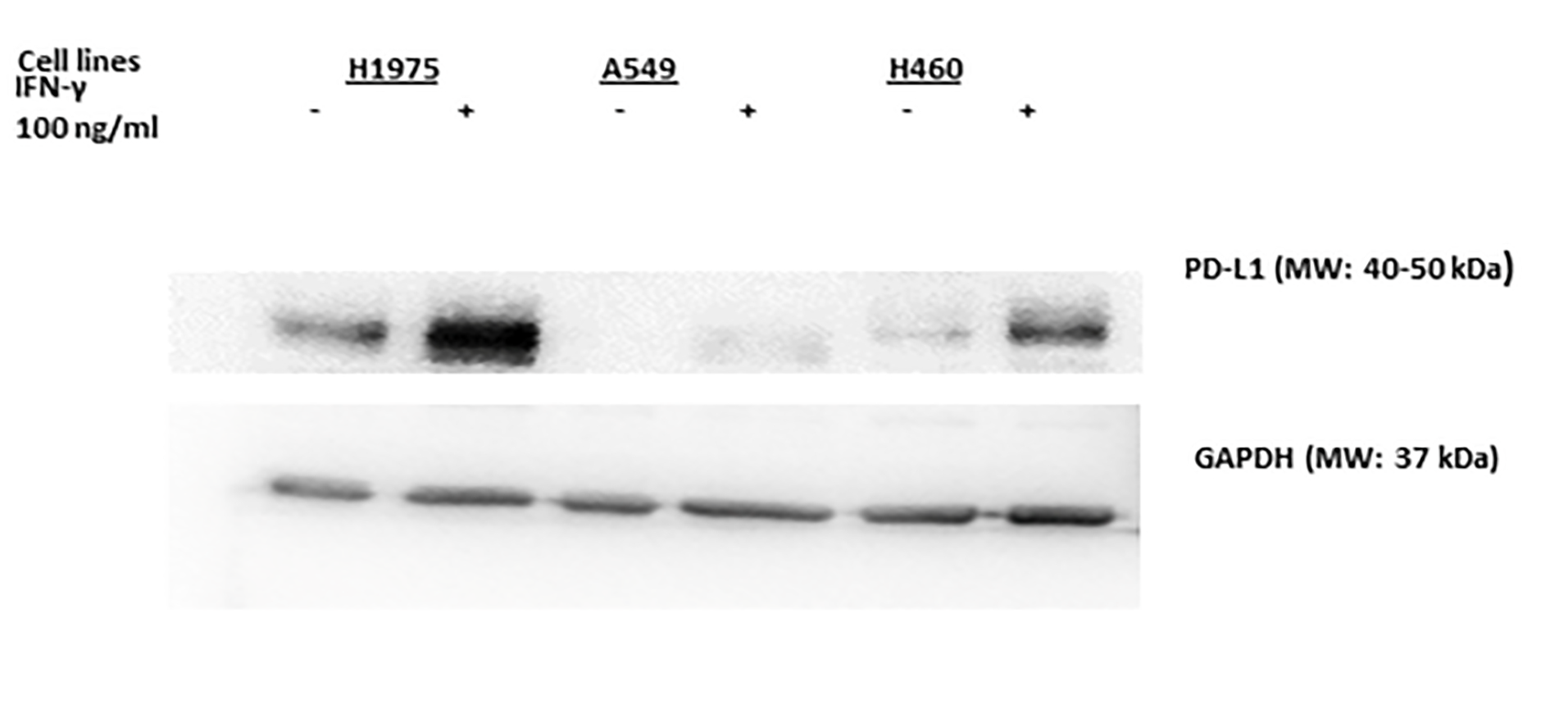

Supplement: Supplementary file 1 — Fig. S1. PD‐L1 expression in NSCLC cell lines before and after induction with IFN‐γ by western blot. Representative western blot of three independent experiments. Total lysate (40μg) of NSCLC cell lines was electrophoresed by SDS‐PAGE (7.5% gel) and immunoblotted for total PD‐L1. Western blot showing strong baseline expression of PD‐L1 in H1975 NSCLC cells, medium in H460 and low expression in A549 cells. Treatment with 100 ng/ml interferon‐γ (IFN‐γ) for 24h upregulated PD‐L1 expression. The rabbit monoclonal antibody E1L3N was used to detect endogenous levels of total PD‐L1 protein and GAPDH served as a loading control. [file MOL2-17-792-s002.tiff]

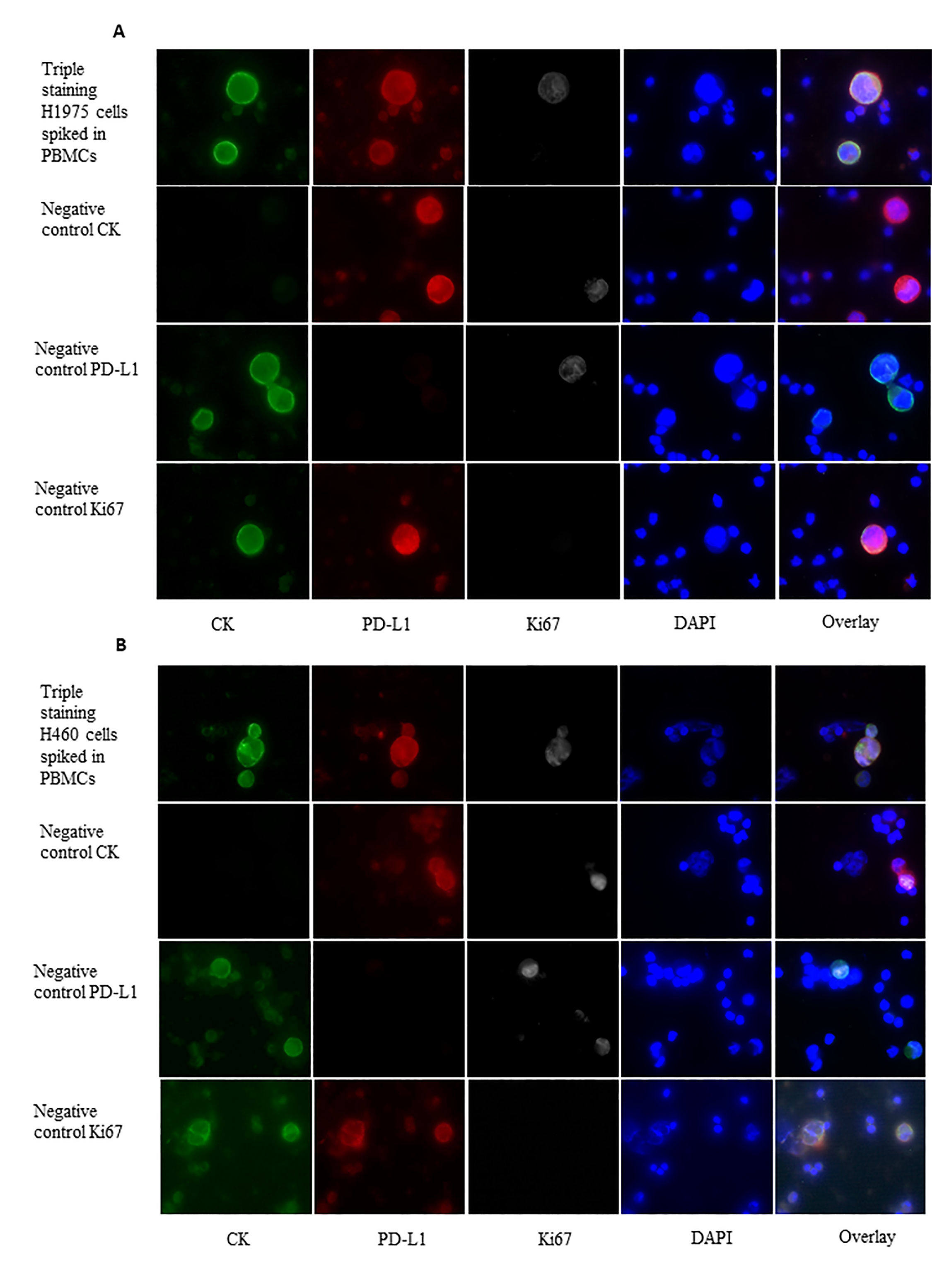

Supplement: Supplementary file 2 — Fig. S2. Expression of CK, PD‐L1 and Ki67 on H1975 (A) and H460 cells (B) spiked in peripheral blood mononuclear cells (PBMCs), by triple immunofluorescence staining. Cytospins of NSCLC cell lines spiked in PBMCs were used as positive and negative controls to evaluate the specificity of the antibodies used for the immunofluorescence staining. Cells were triple stained with pancytokeratin (CK) mouse antibody/secondary anti‐mouse FITC (green), anti‐PD‐L1 rabbit/ secondary anti‐rabbit Alexa Fluor 555 (red) and anti‐Ki67 rabbit 647‐conjugated antibody (grey). Cell nuclei were stained with DAPI (blue). The positive nuclear dotted staining (grey) was evaluated for Ki67 staining. Images were obtained using LASX, (x40). [file MOL2-17-792-s008.tiff]

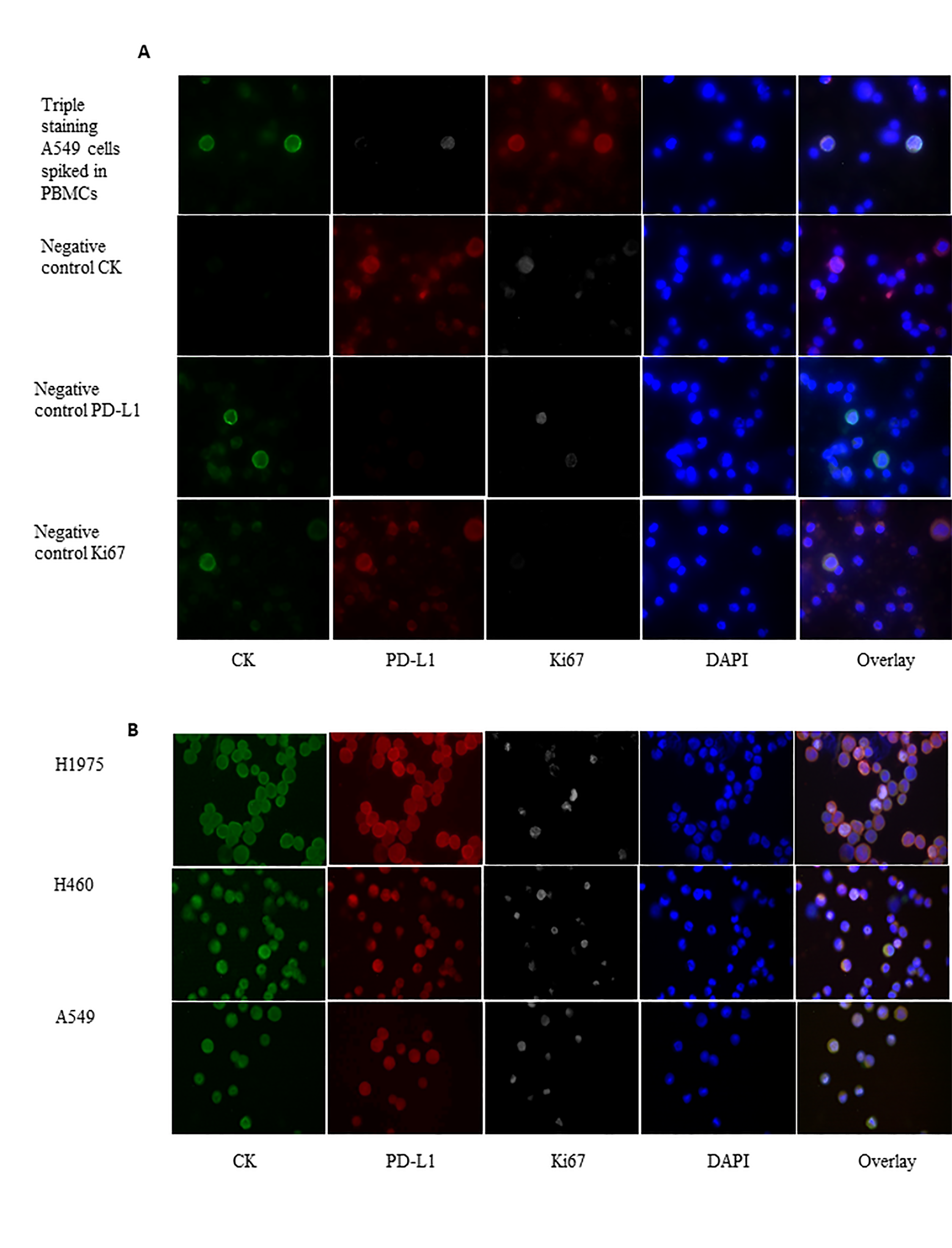

Supplement: Supplementary file 3 — Fig. S3. Expression of CK, PD‐L1 and Ki67 on A549 cells spiked in peripheral blood mononuclear cells (PBMCs) (A) and on NSCLC cell lines (B) by triple immunofluorescence staining. Cytospins of A549 cells spiked in PBMCs were used as positive and negative controls to examine the specificity of the antibodies used, whereas cytospins of H1975, A549 and H460 cells were used for the semi‐quantitative analysis of PD‐L1 expression. Cells were triple stained with pancytokeratin (CK) mouse antibody/secondary anti‐mouse FITC (green), anti‐PD‐L1 rabbit/ secondary anti‐rabbit Alexa Fluor 555 (red) and anti‐Ki67 rabbit 647‐conjugated antibody (grey). Cell nuclei were stained with DAPI (blue). The positive nuclear dotted staining (grey) was evaluated for Ki67 staining. Images were obtained using LASX, (x40). [file MOL2-17-792-s009.tiff]

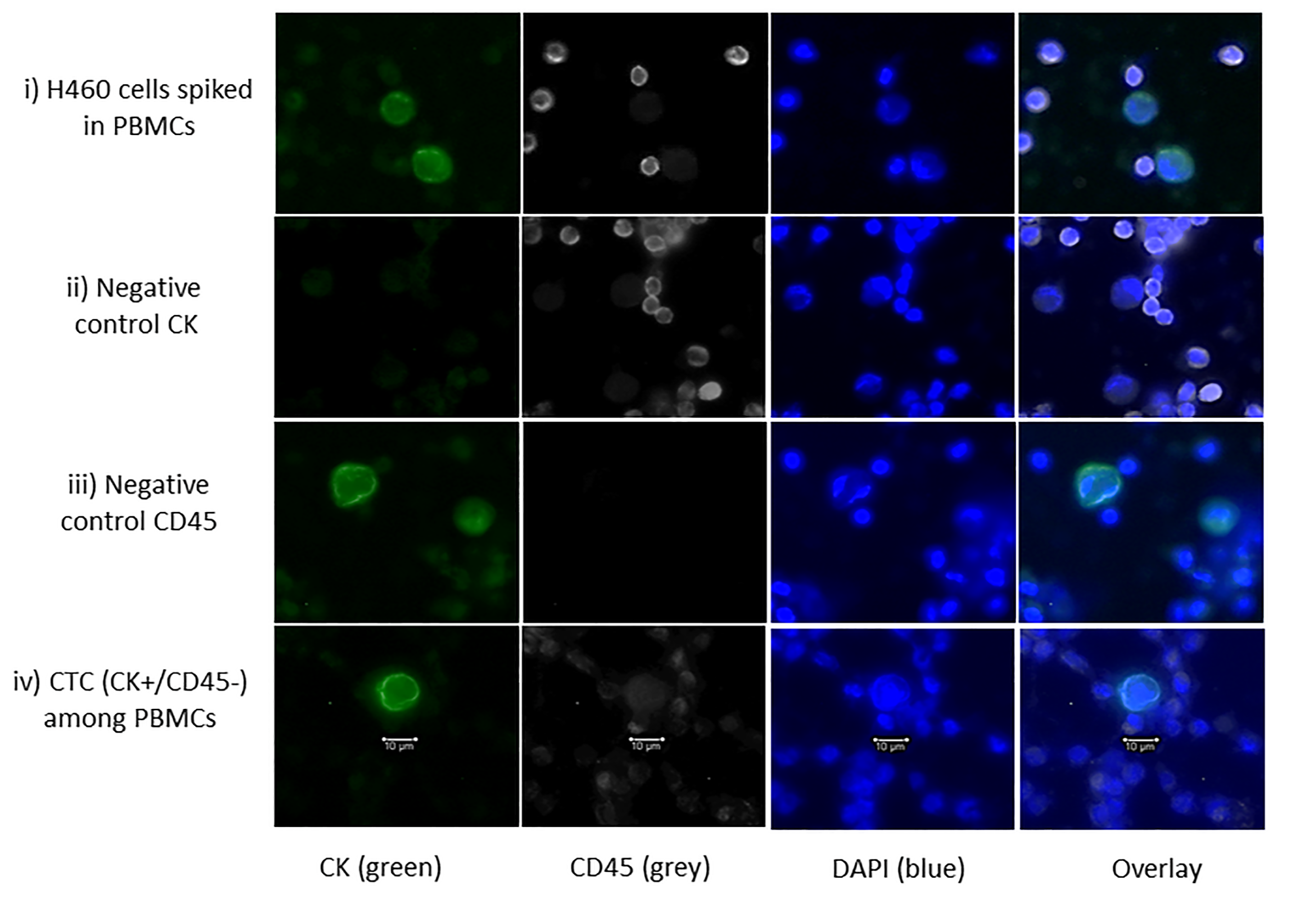

Supplement: Supplementary file 4 — Fig. S4. Double immunofluorescence staining with CK and CD45 antibodies on H460 cells spiked in peripheral blood mononuclear cells (PBMCs) and on PBMCs of NSCLC patients with high circulating tumor cell (CTC) number. (i–iii) Cytospins of H460 cells spiked in PBMCs were used as positive and negative controls to examine the specificity of CK and CD45 antibodies. Cells were double stained with pancytokeratin (CK) mouse antibody/secondary anti‐mouse FITC (green) and anti‐CD45, Alexafluor‐647 conjugated (grey). (iv) Representative image of a CK+/ CD45‐ cell among PBMCs from a patient with high CTC number. Images were obtained using LASX, (x40). [file MOL2-17-792-s006.tiff]

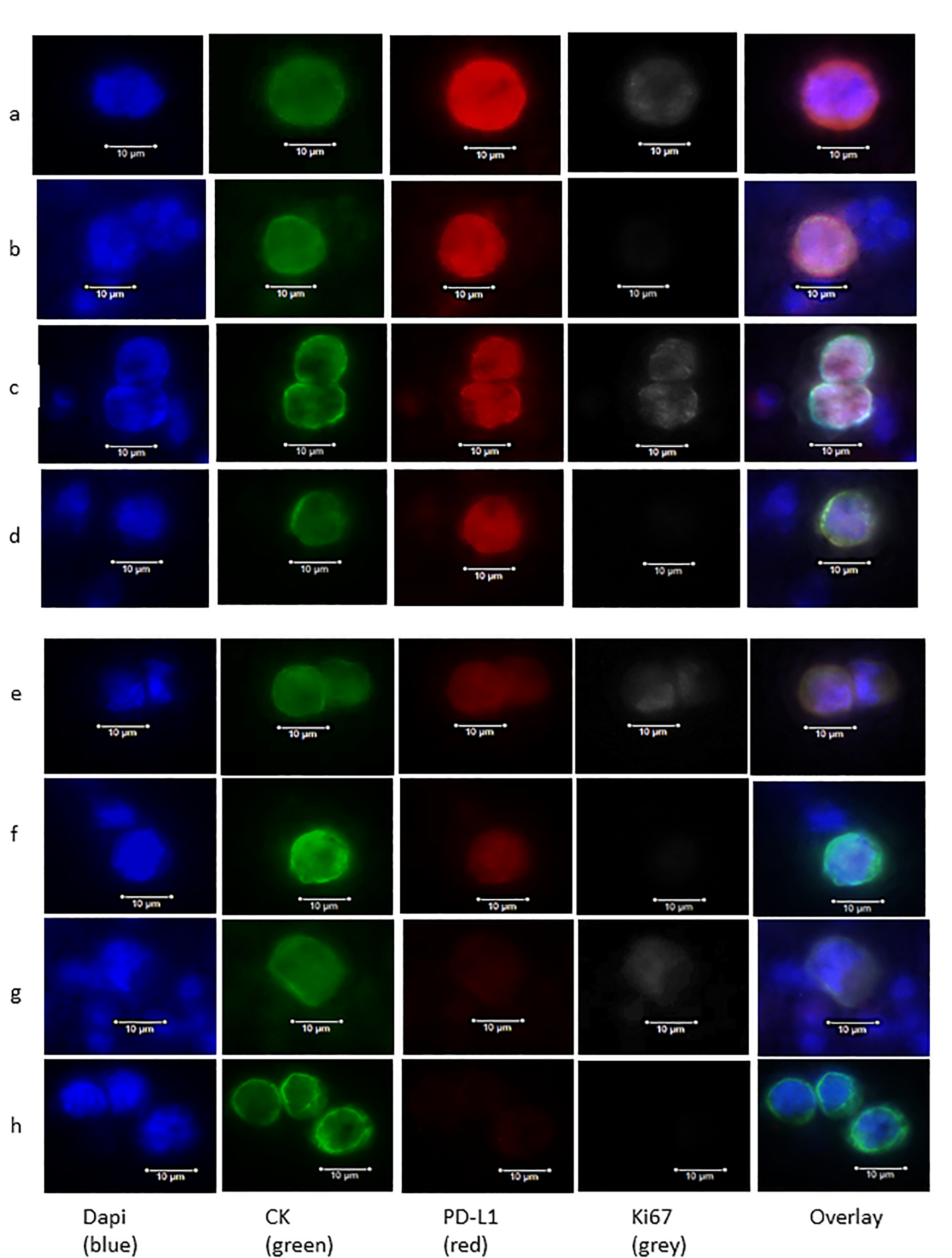

Supplement: Supplementary file 6 — Fig. S6. Expression of CK, PD‐L1 and Ki67 on circulating tumor cells (CTCs) of NSCLC patients by triple immunofluorescence assay. Representative images of phenotypically different CTC subpopulations. Different scenarios of PD‐L1 and Ki67 differential expression in CTCs are shown in panels: (a) PD‐L1high and Ki67+ CTC, (b) PD‐L1high and Ki67‐ CTC among peripheral blood mononuclear cells (PBMCs), (c) two PD‐L1med and Ki67+ CTCs among PBMCs, (d) PD‐L1med and Ki67‐ CTC among PBMCs, (e) PD‐L1low and Ki67+ CTCs, (f) PD‐L1low and Ki67‐ CTC beside PBMCs, (g) PD‐L1neg and Ki67+ CTC among PBMCs and (h) three PD‐L1neg and Ki67‐ CTCs. Cell nuclei were stained with DAPI (blue), Ki67+ CTCs are shown with grey. Images were obtained using LASX, a Leica imaging and analysis system (x63). [file MOL2-17-792-s007.tiff]

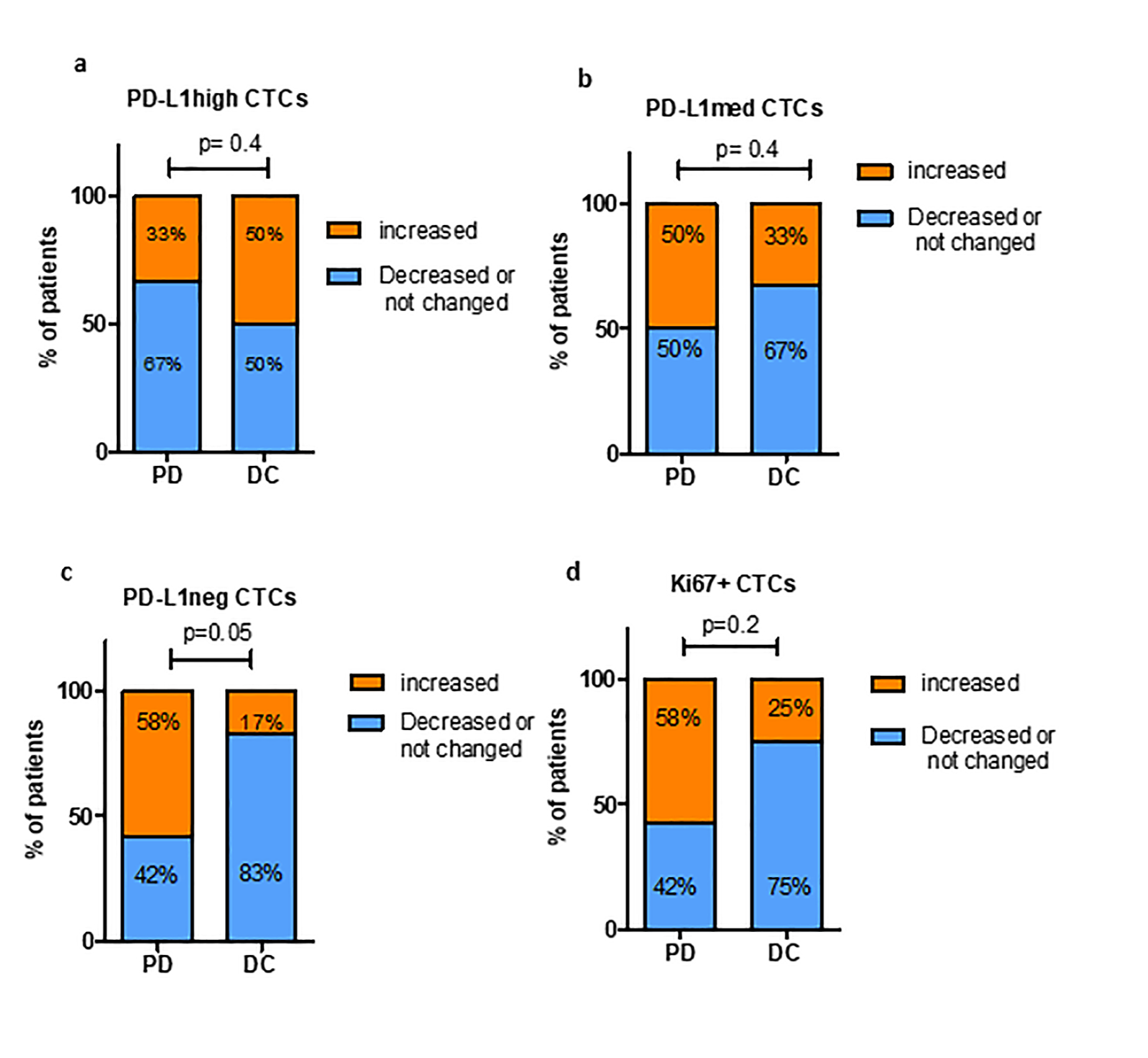

Supplement: Supplementary file 7 — Fig. S7. Changes in circulating tumor cell (CTC) status before and after first cycle, according to PD‐L1high, PD‐L1med, PD‐L1neg and Ki67+ CTCs between progression disease (PD) and disease control (DC) patients. The increased vs decreased or not changed of each CTC status was compared between PD and DC patients. No significant differences were observed in (a) PD‐L1high (b) PD‐L1med, (c) PD‐L1neg and (d) Ki67+ CTCs between PD and DC patients. P‐value was calculated by Fisher's exact test. [file MOL2-17-792-s004.tiff]
