# Supplementary material for: Dynamic monitoring of PD‐L1 and Ki67 in circulating tumor cells of metastatic non‐small cell lung cancer patients treated with pembrolizumab
Source: Mol Oncol. 2022 Dec 16;17(5):792–809. doi: 10.1002/1878-0261.13317 (PMC10158784; doi:10.1002/1878-0261.13317)
Supplement: Supplementary file 8 — Table S1. Circulating tumor cell (CTC) enumeration and characterization according to PD‐L1 and Ki67 in 47 NSCLC patients during pembrolizumab treatment. CTC evaluation according to PD‐L1 and Ki67 in NSCLC patients: (i) at baseline, n=47 patients (ii) post‐first cycle, n=43 (iii) post‐third cycle, n=23 and (iv) at primary resistance, n=19 patients with disease progression at first evaluation of treatment (n/a: not applicable, n/s: no sample, “‐”: no disease progression at first evaluation). Patient's response according to RECIST criteria (PR: partial response, SD: stable disease and PD: progression disease). Results are expressed as CTCs/6x106 PBMCs. [file MOL2-17-792-s005.pdf]

		Number of CTCs/ 6x 10^6 PBMCs							
**Patients n=47**	**Disease Status (PR,SD, PD)**	**Baseline, n=47**		**Post-1st, n=43**		**Post-3rd, n=23**		**Primary-resistance, n=19**	
		**Total CTCs<br>PD-L1high<br>PD-L1med<br>PD-L1low<br>PD-L1neg**	**Total CTCs<br>Ki67+<br>Ki67-**	**Total CTCs<br>PD-L1high<br>PD-L1med<br>PD-L1low<br>PD-L1neg**	**Total CTCs<br>Ki67+<br>Ki67-**	**Total CTCs<br>PD-L1high<br>PD-L1med<br>PD-L1low<br>PD-L1neg**	**Total CTCs<br>Ki67+<br>Ki67-**	**Total CTCs<br>PD-L1high<br>PD-L1med<br>PD-L1low<br>PD-L1neg**	**Total CTCs<br>Ki67+<br>Ki67-**
**N1**	**PD**	**9**<br>2(22%)<br>1(11%)<br>1(11%)<br>5(56%)	**9**<br>3(33%)<br>6(67%)	**5**<br>5(100%)<br>0(0%)<br>0(0%)<br>0(0%)	**5**<br>1(20%)<br>4(80%)	**n/a**	**n/a**	**1094**<br>45(4%)<br>454(41%)<br>571(52%)<br>24(2%)	**1094**<br>328(30%)<br>766(70%)
**N2**	**PD**	**34**<br>10(29%)<br>13(38%)<br>10(29%)<br>1(3%)	**34**<br>8(24%)<br>26(76%)	**1022**<br>317(31%)<br>491(48%)<br>184(18%)<br>30(3%)	**1022**<br>634(62%)<br>388(38%)	**n/a**	**n/a**	**0**	**0**
**N3**	**SD**	**0**	**0**	**3**<br>0(0%)<br>0(0%)<br>2(67%)<br>1(33%)	**3**<br>0(0%)<br>3(100%)	**7**<br>4(57%)<br>3(43%)<br>0(0%)<br>0(0%)	**7**<br>1(14%)<br>6(86%)	**–**	**–**
**N4**	**SD**	**6**<br>1(17%)<br>2(33%)<br>3(50%)<br>0(0%)	**6**<br>0(0%)<br>6(100%)	**10**<br>9(90%)<br>1(10%)<br>0(0%)<br>0(0%)	**10**<br>2(20%)<br>8(80%)	**2**<br>1(50%)<br>0(0%)<br>1(50%)<br>0(0%)	2<br>1(50%)<br>1(50%)	**–**	**–**
**N5**	**SD**	**2**<br>0(0%)<br>0(0%)<br>0(0%)	**2**<br>0(0%)<br>2(100%)	**1**<br>0(0%)<br>0(0%)<br>0(0%)	**1**<br>0(0%)<br>1(100%)	**0**	**0**	**–**	**–**

		2(100%)		1(100%)					
**N6**	**SD**	**25**<br>0(0%)<br>1(4%)<br>4(16%)<br>20(80%)	**25**<br>1(4%)<br>24(96%)	**17**<br>0(0%)<br>0(0%)<br>0(0%)<br>17(100%)	**17**<br>0(0%)<br>17(100%)	**n/a**	**n/a**	**_**	**_**
**N7**	**PD**	**5**<br>0(0%)<br>1(20%)<br>3(60%)<br>1(20%)	**5**<br>2(40%)<br>3(60%)	**7**<br>0(0%)<br>1(14%)<br>5(71%)<br>1(14%)	**7**<br>4(57%)<br>3(43%)	**n/a**	**n/a**	**2**<br>0(0%)<br>1(50%)<br>1(50%)<br>0(0%)	**2**<br>1(50%)<br>1(50%)
**N8**	**SD**	**22**<br>19(86%)<br>3(14%)<br>0(0%)<br>0(0%)	**22**<br>17(77%)<br>5(23%)	**5**<br>3(60%)<br>2(40%)	**5**<br>5(100%)<br>0(0%)	**n/a**	**n/a**	**_**	**_**
**N9**	**PR**	**1**<br>0(0%)<br>0(0%)<br>1(100%)<br>0(0%)	**1**<br>0(0%)<br>1(100%)	**27**<br>0(0%)<br>0(0%)<br>18(67%)<br>9(33%)	**27**<br>1(4%)<br>26(96%)	**2**<br>0(0%)<br>0(0%)<br>2(100%)<br>0(0%)	**2**<br>0(0%)<br>2(100%)	**_**	**_**
**N10**	**PD**	**8**<br>2(25%)<br>1(13%)<br>2(25%)<br>3(38%)	**8**<br>4(50%)<br>4(50%)	**n/s**	**n/s**	**1**<br>0(0%)<br>1(100%)	**1**<br>0(0%)<br>1(100%)	**n/s**	**n/s**
**N11**	**PD**	**1**<br>0(0%)<br>1(100%)<br>0(0%)<br>0(0%)	**1**<br>1(100%)<br>0(0%)	**0**	**0**	**2**<br>0(0%)<br>0(0%)<br>2(100%)<br>0(0%)	**2**<br>0(0%)<br>2(100%)	**n/s**	**n/s**
**N12**	**PD**	**1**<br>1(100%)	**1**<br>0(0%)	**1**<br>0(0%)	**1**<br>1(100%)	**n/a**	**n/a**	**2**<br>0(0%)	**2**<br>2(100%)

		0(0%)<br>0(0%)<br>0(0%)	1(100%)	0(0%)<br>1(100%)<br>0(0%)	0(0%)			1(50%)<br>1(50%)<br>0(0%)	0(0%)
**N13**	**PD**	**2**<br>0(0%)<br>0(0%)<br>1(50%)<br>1(50%)	**2**<br>1(50%)<br>1(50%)	**11**<br>1(9%)<br>0(0%)<br>3(27%)<br>7(64%)	**11**<br>8(73%)<br>3(27%)	**n/a**	**n/a**	**9**<br>0(0%)<br>0(0%)<br>1(11%)<br>8(89%)	**9**<br>7(78%)<br>2(22%)
**N14**	**PD**	**65**<br>49(75%)<br>13(20%)<br>3(5%)<br>0(0%)	**65**<br>32(49%)<br>33(51%)	**0**	**0**	**n/a**	**n/a**	**1**<br>0(0%)<br>0(0%)<br>1(100%)<br>0(0%)	**1**<br>0(0%)<br>1(100%)
**N15**	**SD**	**3**<br>2(67%)<br>1(33%)<br>0(0%)<br>0(0%)	**3**<br>0(0%)<br>3(100%)	**2**<br>0(0%)<br>0(0%)<br>0(0%)<br>2(100%)	**2**<br>0(0%)<br>2(100%)	**10**<br>0(0%)<br>1(10%)<br>0(0%)<br>9(90%)	**10**<br>0(0%)<br>10(100%)	**–**	**–**
**N16**	**PD**	**3**<br>1(33%)<br>1(33%)<br>1(33%)<br>0(%)	**3**<br>2(67%)<br>1(33%)	**5**<br>0(0%)<br>4(80%)<br>0(0%)<br>1(20%)	**5**<br>1(20%)<br>4(80%)	**n/a**	**n/a**	**654**<br>0(0%)<br>117(18%)<br>190(29%)<br>347(53%)	**654**<br>275(42%)<br>379(58%)
**N17**	**PD**	**3**<br>0(%)<br>0(%)<br>0(%)<br>3(100%)	**3**<br>0(%)<br>3(100%)	**0**	**0**	**n/a**	**n/a**	**50**<br>0(%)<br>0(%)<br>0(%)<br>50(100%)	**50**<br>5(10%)<br>45(90%)
**N18**	**SD**	**1**<br>1(100%)<br>0(%)<br>0(%)<br>0(%)	**1**<br>0(%)<br>1(100%)	**2**<br>0(0%)<br>0(0%)<br>1(50%)<br>1(50%)	**2**<br>0(0%)<br>2(100%)	**0**	**0**	**–**	**–**

**N19**	**PD**	**2**<br>0(%)<br>0(%)<br>0(%)<br>2(100%)	**2**<br>0(0%)<br>2(100%)	**7**<br>1(14%)<br>3(43%)<br>3(43%)<br>0(0%)	**7**<br>3(43%)<br>4(57%)	**n/a**	**n/a**	**0**	**0**
**N20**	**PD**	**4**<br>0(0%)<br>0(0%)<br>0(0%)<br>4(100%)	**4**<br>0(%)<br>4(100%)	**n/a**	**n/a**	**n/a**	**n/a**	**n/s**	**n/s**
**N21**	**SD**	**1**<br>0(0%)<br>1(100%)<br>0(0%)<br>0(0%)	**1**<br>0(%)<br>1(100%)	**2**<br>0(0%)<br>1(50%)<br>0(0%)<br>1(50%)	**2**<br>0(0%)<br>2(100%)	**9**<br>0(0%)<br>0(0%)<br>4(44%)<br>5(56%)	**9**<br>1(11%)<br>8(89%)	**–**	**–**
**N22**	**SD**	**1**<br>0(0%)<br>1(100%)<br>0(0%)<br>0(0%)	**1**<br>0(%)<br>1(100%)	**1**<br>0(0%)<br>1(100%)<br>0(0%)<br>0(0%)	**1**<br>1(100%)<br>0(0%)	**2**<br>0(0%)<br>0(0%)<br>0(0%)<br>2(100%)	**2**<br>0(0%)<br>2(100%)	**–**	**–**
**N23**	**SD**	**90**<br>1(1%)<br>16(18%)<br>35(39%)<br>38(42%)	**90**<br>18(20%)<br>72(80%)	**3**<br>0(0%)<br>1(33%)<br>0(0%)<br>2(67%)	**3**<br>3(100%)<br>0(0%)	**n/a**	**n/a**	**–**	**–**
**N24**	**SD**	**6**<br>1(17%)<br>2(33%)<br>1(17%	**6**<br>1(17%)<br>5(83%)	**0**	**0**	**2**<br>2(100%)<br>0(0%)<br>0(0%)<br>0(0%)	**2**<br>0(0%)<br>2(100%)	**–**	**–**
**N25**	**PD**	**3**	**3**	**n/a**	**n/a**	**n/a**	**n/a**	**42**	**42**

		0(0%)<br>1(33%)<br>1(33%)<br>1(33%)	1(33%)<br>2(67%)					0(%)<br>0(%)<br>0(%)<br>42(100%)	0(%)<br>42(100%)
**N26**	**PD**	**2**<br>2(100%)<br>0(0%)<br>0(0%)<br>0(0%)	**2**<br>1(50%)<br>1(50%)	**1**<br>0(0%)<br>0(0%)<br>1(100%)<br>0(0%)	**1**<br>0(0%)<br>1(100%)	**n/a**	**n/a**	**2**<br>1(50%)<br>0(%)<br>0(%)<br>1(50%)	**2**<br>1(50%)<br>1(50%)
**N27**	**SD**	**0**	**0**	**1**<br>1(100%)<br>0(0%)<br>0(0%)<br>0(0%)	**1**<br>0(0%)<br>1(100%)	**1**<br>0(0%)<br>0(0%)<br>1(100%)<br>0(0%)	**1**<br>0(0%)<br>1(100%)	**–**	**–**
**N28**	**SD**	**1**<br>1(100%)<br>0(0%)<br>0(0%)<br>0(0%)	**1**<br>0(0%)<br>1(100%)	**1**<br>0(0%)<br>1(100%)<br>0(0%)<br>0(0%)	**1**<br>1(100%)<br>0(0%)	**2**<br>0(0%)<br>1(50%)<br>0(0%)<br>1(50%)	**2**<br>1(50%)<br>1(50%)	**–**	**–**
**N29**	**SD**	**1**<br>0(0%)<br>0(0%)<br>0(0%)<br>1(100%)	**1**<br>0(0%)<br>1(100%)	**3**<br>2(67%)<br>0(0%)<br>0(0%)<br>1(33%)	**3**<br>2(67%)<br>1(33%)	**0**	**0**	**–**	**–**
**N30**	**PR**	**5**<br>0(0%)<br>3(60%)<br>2(40%)<br>0(0%)	**5**<br>1(20%)<br>4(80%)	**32**<br>20(63%)<br>10(31%)<br>2(6%)<br>0(0%)	**32**<br>5(16%)<br>27(84%)	**3**<br>0(0%)<br>0(0%)<br>3(100%)<br>0(0%)	**3**<br>0(0%)<br>3(100%)	**–**	**–**
**N31**	**SD**	**1**<br>0(0%)<br>0(0%)	**1**<br>0(0%)<br>1(100%)	**0**	**0**	**0**	**0**	**–**	**–**

		1(100%)<br>0(0%)							
**N32**	**PD**	**3**<br>2(67%)<br>1(33%)<br>0(0%)<br>0(0%)	**3**<br>1(33%)<br>2(67%)	**5**<br>2(40%)<br>2(40%)<br>1(20%)<br>0(0%)	**5**<br>0(0%)<br>5(100%)	**n/a**	**n/a**	**2**<br>0(0%)<br>0(0%)<br>2(100%)<br>0(0%)	**2**<br>1(50%)<br>1(50%)
**N33**	**SD**	**1**<br>1(100%)<br>0(0%)<br>0(0%)<br>0(0%)	**1**<br>1(100%)<br>0(0%)	**2**<br>0(0%)<br>1(50%)<br>0(0%)<br>1(50%)	**2**<br>0(0%)<br>2(100%)	**6**<br>3(50%)<br>3(50%)<br>0(0%)<br>0(0%)	**6**<br>1(17%)<br>5(83%)	_	_
**N34**	**SD**	**3**<br>0(0%)<br>1(33%)<br>2(67%)<br>0(0%)	**3**<br>0(0%)<br>3(100%)	**8**<br>2(25%)<br>2(25%)<br>3(38%)<br>1(13)	**8**<br>0(0%)<br>8(100%)	**0**	**0**	_	_
**N35**	**PD**	**2**<br>2(100%)<br>0(0%)<br>0(0%)<br>0(0%)	**2**<br>1(50%)<br>1(50%)	**n/a**	**n/a**	**n/a**	**n/a**	**15**<br>0(0%)<br>2(13%)<br>6(40%)<br>7(47%)	**15**<br>3(20%)<br>12(80%)
**N36**	**PD**	**150**<br>45(30%)<br>84(56%)<br>21(14%)<br>0(0%)	**150**<br>58(39%)<br>92(61%)	**0**	**0**	**n/a**	**n/a**	**3**<br>1(33%)<br>0(0%)<br>2(67%)<br>0(0%)	**3**<br>2(67%)<br>1(33%)
**N37**	**SD**	**9**<br>1(11%)<br>0(0%)<br>5(56%)<br>3(33%)	**9**<br>1(11%)<br>8(89%)	**23**<br>14(61%)<br>6(26%)<br>3(13%)<br>0(0%)	**23**<br>5(22%)<br>18(78%)	**n/a**	**n/a**	_	_
**N38**	**SD**	**99**	**99**	**5**	**5**	**9**	**9**	_	_

		0(0%)<br>0(0%)<br>20(20%)<br>79(80%)	5(5%)<br>94(95%)	0(0%)<br>1(20%)<br>2(40%)<br>2(40%)	2(40%)<br>3(60%)	8(89%)<br>0(0%)<br>1(11%)<br>0(0%)	3(33%)<br>6(67%)		
**N39**	**PD**	**2**<br>2(100%)<br>0(0%)<br>0(0%)<br>0(0%)	**2**<br>1(50%)<br>1(50%)	**4**<br>1(25%)<br>0(0%)<br>2(50%)<br>1(25%)	**4**<br>0(0%)<br>4(100%)	**n/a**	**n/a**	**31**<br>6(19%)<br>16(52%)<br>8(26%)<br>8(26%)	**31**<br>3(10%)<br>28(90%)
**N40**	**SD**	**1**<br>1(100%)<br>0(0%)<br>0(0%)<br>0(0%)	**1**<br>1(100%)<br>0(0%)	**1**<br>0(0%)<br>1(100%)<br>0(0%)<br>0(0%)	**1**<br>0(0%)<br>1(100%)	**0**	**0**	**–**	**–**
**N41**	**PD**	**4**<br>0(0%)<br>0(0%)<br>0(0%)<br>4(100%)	**4**<br>0(0%)<br>4(100%)	**2**<br>0(0%)<br>0(0%)<br>0(0%)<br>2(100%)	**2**<br>2(100%)<br>0(0%)	**n/a**	**n/a**	**2**<br>0(0%)<br>0(0%)<br>0(0%)<br>2(100%)	**2**<br>0(0%)<br>2(100%)
**N42**	**SD**	**2**<br>0(0%)<br>0(0%)<br>0(0%)<br>2(100%)	**2**<br>0(0%)<br>2(100%)	**0**	**0**	**8**<br>0(0%)<br>0(0%)<br>8(100%)<br>0(0%)	**8**<br>0(0%)<br>8(100%)	**–**	**–**
**N43**	**PD**	**0**	**0**	**126**<br>1(1%)<br>22(17%)<br>53(42%)<br>50(40%)	**126**<br>16(13%)<br>110(87%)	**n/a**	**n/a**	**3**<br>0(0%)<br>0(0%)<br>1(33%)<br>2(67%)	**3**<br>3(67%)<br>1(33%)
**N44**	**SD**	**33**<br>0(0%)<br>1(3%)<br>7(21%)	**33**<br>9(27%)<br>24(73%)	**4**<br>0(0%)<br>1(25%)<br>2(50%)	**4**<br>1(25%)<br>3(75%)	**41**<br>0(0%)<br>1(2%)<br>23(56%)	**41**<br>3(7%)<br>38(93%)	**–**	**–**

		25(76%)		1(25%)		17(41%)			
**N45**	**SD**	**88**<br>3(3%)<br>9(10%)<br>26(30%)<br>50(57%)	**88**<br>20(23%)<br>68(77%)	**13**<br>4(31%)<br>2(15%)<br>2(15%)<br>5(38%)	**13**<br>8(62%)<br>5(38%)	**6**<br>0(0%)<br>1(17%)<br>2(33%)<br>3(50%)	**6**<br>0(0%)<br>6(100%)	**_**	**_**
**N46**	**SD**	**39**<br>1(3%)<br>7(18%)<br>8(21%)<br>23(59%)	**39**<br>27(69%)<br>12(31%)	**4**<br>2(50%)<br>1(25%)<br>0(0%)<br>1(25%)	**4**<br>4(100%)<br>0(0%)	**n/a**	**n/a**	_	**_**
**N47**	**PD**	**22**<br>1(5%)<br>1(5%)<br>4(18%)<br>16(73%)	**22**<br>13(59%)<br>9(41%)	**65**<br>1(2%)<br>5(8%)<br>12(18%)<br>47(72%)	**65**<br>18(28%)<br>47(72%)	**n/a**	**n/a**	**n/s**	**n/s**

Supplementary Table 1. CTC evaluation according to PD-L1 and Ki67 in NSCLC patients treated with Pembrolizumab n=47, i) at baseline, n=47 ii) post-first cycle, n=43 iii) post-third cycle, n=23 and iv) at primary resistance, n=19 patients with disease progression at first evaluation of treatment (n/a: not applicable, n/s: no sample, "-": no disease progression at first evaluation). Patient's response according to RECIST criteria (PR: partial response, SD: stable disease and PD: progression disease). Results are expressed as CTCs/6x10^6 PBMCs.
