# Supplementary material for: Dynamic monitoring of PD‐L1 and Ki67 in circulating tumor cells of metastatic non‐small cell lung cancer patients treated with pembrolizumab
Source: Mol Oncol. 2022 Dec 16;17(5):792–809. doi: 10.1002/1878-0261.13317 (PMC10158784; doi:10.1002/1878-0261.13317)
Supplement: Supplementary file 9 — Appendix S1 [file MOL2-17-792-s001.docx]

**Appendix S1.** Legends of supplementary figures and table.

**Supplementary Table 1. Circulating tumor cell (CTC) enumeration and characterization according to PD-L1 and Ki67 in 47 NSCLC patients during pembrolizumab treatment.** CTC evaluation according to PD-L1 and Ki67 in NSCLC patients: i) at baseline, n=47 patients ii) post-first cycle, n=43 iii) post-third cycle, n=23 and iv) at primary resistance, n=19 patients with disease progression at first evaluation of treatment (n/a: not applicable, n/s: no sample, “-”: no disease progression at first evaluation). Patient’s response according to RECIST criteria (PR: partial response, SD: stable disease and PD: progression disease). Results are expressed as CTCs/6x10^6^ PBMCs.

**Figure S1. PD-L1 expression in NSCLC cell lines before and after induction with IFN-γ by western blot.** Representative western blot of three independent experiments. Total lysate (40μg) of NSCLC cell lines was electrophoresed by SDS-PAGE (7.5% gel) and immunoblotted for total PD-L1. Western blot showing strong baseline expression of PD-L1 in H1975 NSCLC cells, medium in H460 and low expression in A549 cells. Treatment with 100 ng/ml interferon-γ (IFN-γ) for 24h upregulated PD-L1 expression. The rabbit monoclonal antibody E1L3N was used to detect endogenous levels of total PD-L1 protein and GAPDH served as a loading control.

**Figure S2.** **Expression of CK, PD-L1 and Ki67 on H1975 (A) and H460 cells (B) spiked in peripheral blood mononuclear cells (PBMCs), by triple immunofluorescence staining.** Cytospins of NSCLC cell lines spiked in PBMCs were used as positive and negative controls to evaluate the specificity of the antibodies used for the immunofluorescence staining. Cells were triple stained with pancytokeratin (CK) mouse antibody/secondary anti-mouse FITC (green), anti-PD-L1 rabbit/ secondary anti-rabbit Alexa Fluor 555 (red) and anti-Ki67 rabbit 647-conjugated antibody (grey). Cell nuclei were stained with DAPI (blue). The positive nuclear dotted staining (grey) was evaluated for Ki67 staining. Images were obtained using LASX, (x40).

**Figure S3.** **Expression of CK, PD-L1 and Ki67 on A549 cells spiked in peripheral blood mononuclear cells (PBMCs) (A) and on NSCLC cell lines (B) by triple immunofluorescence staining**. Cytospins of A549 cells spiked in PBMCs were used as positive and negative controls to examine the specificity of the antibodies used, whereas cytospins of H1975, A549 and H460 cells were used for the semi-quantitative analysis of PD-L1 expression. Cells were triple stained with pancytokeratin (CK) mouse antibody/secondary anti-mouse FITC (green), anti-PD-L1 rabbit/ secondary anti-rabbit Alexa Fluor 555 (red) and anti-Ki67 rabbit 647-conjugated antibody (grey). Cell nuclei were stained with DAPI (blue). The positive nuclear dotted staining (grey) was evaluated for Ki67 staining. Images were obtained using LASX, (x40).

**Figure S4.** **Double immunofluorescence staining with CK and CD45 antibodies on H460 cells spiked in peripheral blood mononuclear cells (PBMCs) and on PBMCs of NSCLC patients with high circulating tumor cell (CTC) number.** i-iii) Cytospins of H460 cells spiked in PBMCs were used as positive and negative controls to examine the specificity of CK and CD45 antibodies. Cells were double stained with pancytokeratin (CK) mouse antibody/secondary anti-mouse FITC (green) and anti-CD45, Alexafluor-647 conjugated (grey). iv) Representative image of a CK+/ CD45- cell among PBMCs from a patient with high CTC number. Images were obtained using LASX, (x40).

**Figure S5.** **Setting-up the thresholds in circulating tumor cells based on three NSCLC cell lines with different PD-L1 expression levels**. The mean fluorescence intensity per pixel (MFI) of PD-L1 expression in A549, H460 and H1975 cell lines was determined by imaging and analysis system LASX (Leica, microsystems). The mean intensity (m) of PD-L1 staining in each cell line is used to define the thresholds for semi-quantification and it was significantly different among the cell lines tested (*p<0.0001). The highest mean intensity was observed in H1975 cells (mean ± SD; 58 ± 16), a medium intensity of PD-L1 was observed in H460 cells (39 ± 5.5) and the lowest intensity of PD-L1 staining was observed in A549 cells (24 ± 5.3).

**Figure S6.** **Expression of CK, PD-L1 and Ki67 on circulating tumor cells (CTCs) of NSCLC patients by triple immunofluorescence assay.** Representative images of phenotypically different CTC subpopulations. Different scenarios of PD-L1 and Ki67 differential expression in CTCs are shown in panels: (a) PD-L1high and Ki67+ CTC, (b) PD-L1high and Ki67- CTC among peripheral blood mononuclear cells (PBMCs), (c) two PD-L1med and Ki67+ CTCs among PBMCs, (d) PD-L1med and Ki67- CTC among PBMCs, (e) PD-L1low and Ki67+ CTCs, (f) PD-L1low and Ki67- CTC beside PBMCs, (g) PD-L1neg and Ki67+ CTC among PBMCs and (h) three PD-L1neg and Ki67- CTCs. Cell nuclei were stained with DAPI (blue), Ki67+ CTCs are shown with grey. Images were obtained using LASX, a Leica imaging and analysis system (x63).

**Figure S7. Changes in circulating tumor cell (CTC) status before and after first cycle, according to PD-L1high, PD-L1med, PD-L1neg and Ki67+ CTCs between progression disease (PD) and disease control (DC) patients.** The increased vs decreased or not changed of each CTC status was compared between PD and DC patients. No significant differences were observed in (a) PD-L1high (b) PD-L1med, (c) PD-L1neg and (d) Ki67+ CTCs between PD and DC patients. P-value was calculated by Fisher’s exact test.

**CTC analysis between PD and DC patients before and after the first cycle of treatment (n=24)**
